# A hidden Markov model reliably characterizes ketamine-induced spectral dynamics in macaque local field potentials and human electroencephalograms

**DOI:** 10.1371/journal.pcbi.1009280

**Published:** 2021-08-18

**Authors:** Indie C. Garwood, Sourish Chakravarty, Jacob Donoghue, Meredith Mahnke, Pegah Kahali, Shubham Chamadia, Oluwaseun Akeju, Earl K. Miller, Emery N. Brown

**Affiliations:** 1 Harvard-MIT Division of Health Sciences and Technology, Massachusetts Institute of Technology, Cambridge, Massachusetts, United States of America; 2 The Picower Institute for Learning and Memory, Massachusetts Institute of Technology, Cambridge, Massachusetts, United States of America; 3 Department of Brain and Cognitive Sciences, Massachusetts Institute of Technology, Cambridge, Massachusetts, United States of America; 4 Department of Anesthesia, Critical Care, and Pain Medicine, Massachusetts General Hospital, Boston, Massachusetts, United States of America; Ghent University, BELGIUM

## Abstract

Ketamine is an NMDA receptor antagonist commonly used to maintain general anesthesia. At anesthetic doses, ketamine causes high power gamma (25-50 Hz) oscillations alternating with slow-delta (0.1-4 Hz) oscillations. These dynamics are readily observed in local field potentials (LFPs) of non-human primates (NHPs) and electroencephalogram (EEG) recordings from human subjects. However, a detailed statistical analysis of these dynamics has not been reported. We characterize ketamine’s neural dynamics using a hidden Markov model (HMM). The HMM observations are sequences of spectral power in seven canonical frequency bands between 0 to 50 Hz, where power is averaged within each band and scaled between 0 and 1. We model the observations as realizations of multivariate beta probability distributions that depend on a discrete-valued latent state process whose state transitions obey Markov dynamics. Using an expectation-maximization algorithm, we fit this beta-HMM to LFP recordings from 2 NHPs, and separately, to EEG recordings from 9 human subjects who received anesthetic doses of ketamine. Our beta-HMM framework provides a useful tool for experimental data analysis. Together, the estimated beta-HMM parameters and optimal state trajectory revealed an alternating pattern of states characterized primarily by gamma and slow-delta activities. The mean duration of the gamma activity was 2.2s([1.7,2.8]s) and 1.2s([0.9,1.5]s) for the two NHPs, and 2.5s([1.7,3.6]s) for the human subjects. The mean duration of the slow-delta activity was 1.6s([1.2,2.0]s) and 1.0s([0.8,1.2]s) for the two NHPs, and 1.8s([1.3,2.4]s) for the human subjects. Our characterizations of the alternating gamma slow-delta activities revealed five sub-states that show regular sequential transitions. These quantitative insights can inform the development of rhythm-generating neuronal circuit models that give mechanistic insights into this phenomenon and how ketamine produces altered states of arousal.

## 1 Author summary

Monitoring brain activity during anesthesia can provide insights into the underlying mechanisms of how anesthetics elicit altered states of consciousness. Ketamine, a commonly used anesthetic, is known to cause short duration bursts of high frequency electrophysiological activity in the brain, but the neural mechanisms underlying this activity are not well understood. A key limitation in developing accurate models of the underlying mechanism is a lack of detailed knowledge of the dynamic structure and spectral properties of ketamine-induced oscillations. In this work, we address this limitation by developing a statistical framework to quantify ketamine-induced neural activity. Our framework is based on a hidden Markov model, which assumes that the neural activity switches among discrete states, each of which has its own distinctive probabilistic spectral representation. By estimating this versatile statistical model from electrophysiology data, we generated detailed descriptions of the dynamic properties and oscillatory signatures associated with ketamine-induced neurophysiological states in non-human primates and in human patients. Furthermore, we identified additional ketamine-induced states that have not yet been reported. In summary, our detailed quantitative descriptions of ketamine-induced spectra can aid further developments of neurophysiological mechanistic models of ketamine as well as biomarker discovery for clinical anesthesia monitoring.

## 2 Introduction

Ketamine is a phenylcyclidine derivative and one of the most commonly used anesthetics in clinical use world-wide [1–4]. It is classified as a WHO Essential Medicine [5], and is often the only anesthetic available in clinics of developing countries [4]. At low doses, ketamine is known to create a state of “dissociative anesthesia” characterized by altered sensory perception and analgesia [1, 2]. At high doses it also leads to loss of consciousness, and therefore it is often used as an anesthetic agent during surgery in humans and animals [3, 6–8]. Under anesthetic dosages, ketamine produces distinct oscillatory signatures in the electroencephalogram (EEG) of healthy volunteers and patients [9–11]. In a recent retrospective study by Akeju et al. [10], the authors found that the frontal EEG from patients who received ketamine for the induction of general anesthesia showed distinct alternating periods of intermittent high power activity in the 27–40 Hz and 0.1–4 Hz frequency bands. Spontaneous gamma oscillations (i.e. 25–50 Hz) have also been demonstrated under high dose ketamine in mice [12], rats [13], cats [14], non-human primates (NHPs) [15–17], and sheep [18]. Recent neuroscience research in NHPs under high-dose ketamine revealed prominent gamma oscillations modulated by slow wave (0.3 Hz) activity [17], similar to the alternating periods of activity in gamma and slow-delta bands observed in humans [10]. Objective characterization of the intermittent band-limited spectral dynamics induced by ketamine would be useful both for monitoring patients during ketamine-induced unconsciousness in clinical settings, as well as for aiding computational and experimental neuroscience research aimed at building a mechanistic understanding of the phenomena. Therefore, the availability of an efficient analytic tool, which can objectively detect and characterize ketamine-induced transient neural dynamics, can catalyze both clinical and neuroscience innovations.

Ketamine’s primary pharmacodynamic effect is N-Methyl-d-aspartate (NMDA) receptor antagonism [19]. One putative mechanism of ketamine-induced cortical gamma oscillations is that ketamine preferentially blocks NMDA receptors of fast spiking inhibitory gamma aminobutyric acid (GABA) interneurons. The blocking of the NMDA receptors on the interneurons results in reduced interneuron activity and a reduction of GABA release at the synapse between interneurons and excitatory pyramidal neurons. The reduction of GABA causes disinhibition of the pyramidal neurons, resulting in increased pyramidal neuron activity that manifests as cortical gamma oscillations [20, 21]. This mechanism, however, does not explain the presence of periodic high-power gamma activity alternating with slow-delta activity. A recent modeling study suggests a cyclic mechanism where in each cycle, the pyramidal neurons that are disinhibited at the beginning of the cycle provide sufficient input to the interneurons for the latter to overcome the ketamine inhibition and release GABA, which then results in temporary inhibition of the pyramidal neurons towards the end of the cycle [22]. A statistical characterization of the ketamine-induced alternating gamma and slow-delta oscillation activity in neural data can provide informative quantitative constraints for mechanistic neuronal circuit models of ketamine-induced spectral dynamics. Such mechanistic models can further aid the understanding of how ketamine causes altered states of arousal. Examples of how detailed statistical analyses of neural data has guided neurophysiological modeling studies can be found in existing works that studied propofol-induced unconsciousness [23–26].

State-space models provide a versatile statistical framework for analyzing the dynamics of complex neural time-series data [27]. These models have been widely used in neuroscience, from decoding hippocampal place cells [28], to tracking movement intention in the motor cortex [29–31], to assessing the level of unconsciousness in medically-induced coma [32]. A particular class of state-space models, known as Hidden Markov Models (HMMs), have been used to describe underlying dynamical processes driven by stochastic switching between discrete states, where each state is associated with a distinct observation probability distribution [33–38]. HMMs have been used to objectively decode neurophysiological states, including detecting seizure events [39], classifying sleep stages [40–43], decoding speech [44, 45], and decoding movement intention [46, 47]. HMMs have also been used to provide detailed statistical descriptions of neural phenomena [48–50]. Since ketamine-induced cortical activity is characterized by distinct switching patterns in oscillatory dynamics, an HMM is a plausible statistical model for both estimating neurophysiological states and characterizing their statistical properties. Our preliminary analysis using an HMM to characterize ketamine-induced spectral dynamics in human EEG and in local field potential (LFP) from NHPs yielded promising results [51]. Along similar lines, the recent work by Li and Mashour [11], using a particular class of HMMs [52] to characterize multichannel scalp EEG from healthy volunteers, further supports the utility of HMMs to analyze ketamine spectra. By inferring an HMM from neural activity spectra, we can quantitatively characterize the discontinuous spectral dynamics in terms of the parameters of the best-fitting observation and state transition models, as well as the state trajectory corresponding to the entire data sequence.

**Fig 1:**
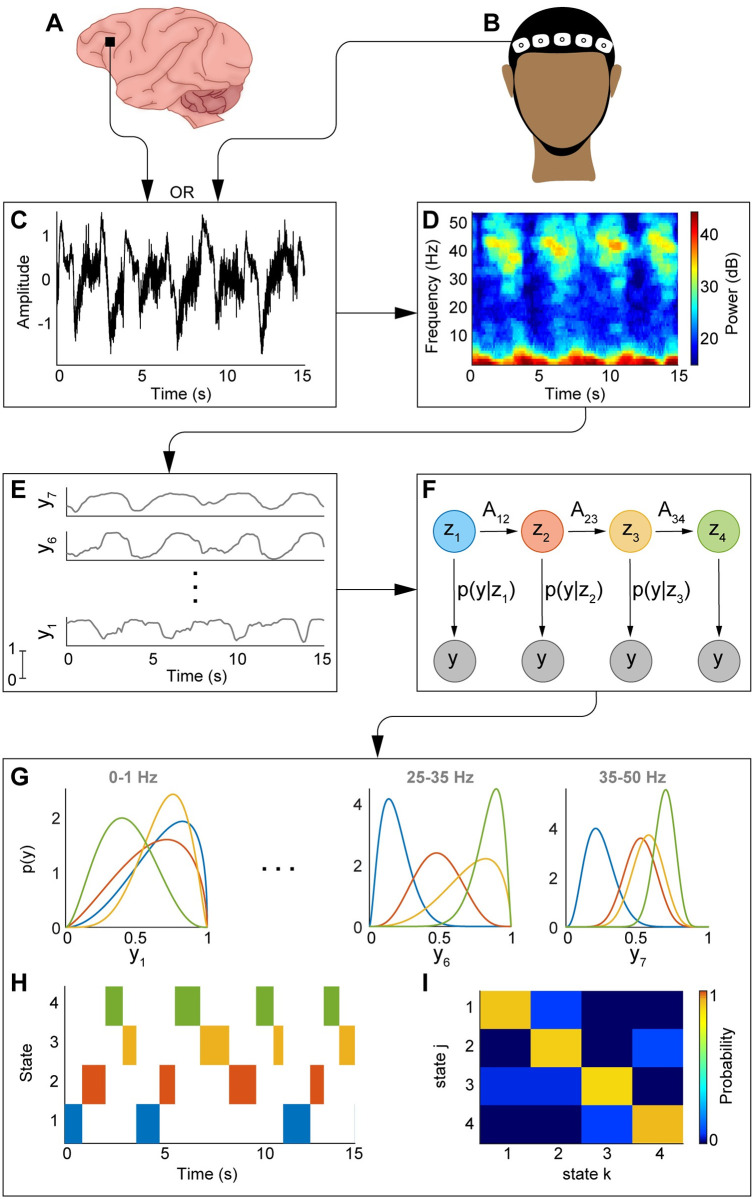
Schematic of the beta-HMM algorithm. Neural recordings from primate LFP (A) or human EEG (B) serve as inputs to the algorithm. Electrical recordings (C) are converted to their time-frequency representation via multitaper spectral analysis (D). The spectral observations are split into 7 frequency bands and scaled between 0 and 1 (E). These observations are then fit to the beta-HMM (F) via the EM algorithm, and the state trajectory is estimated from the Viterbi algorithm. Outputs of the algorithm are the beta observation distributions for each state (G), estimated state trajectory (H), and a transition matrix (I) describing the state switching dynamics.

In this work, we develop an HMM-based analysis framework to characterize ketamine-induced spectral dynamics recorded in neurophysiological data from 2 NHPs and 9 human patients in the operating room (OR) (Fig 1). The observations that go into our HMM analysis are sequences of spectral power in seven frequency bands between 0 and 50 Hz. Neural oscillations are commonly characterized according to canonical frequency bands. In this study, we define these according to those defined by Purdon, et al. [53]: slow (0–1 Hz), delta (1–4 Hz), theta (4–8 Hz), alpha (8–12 Hz), beta (12–25 Hz), low gamma (25–35 Hz), and gamma (35–50 Hz). The power is averaged within each band and scaled between 0 and 1 to facilitate comparison among data sets that vary in signal amplitude but not in the spectral profiles they represent. To describe this multivariate observation sequence, we assume that the spectral dynamics are governed by a discrete-valued latent state process, where each state corresponds to a distinct spectral profile. We model these spectral profiles as realizations of state-specific and frequency-band specific beta distributions. From the estimated HMM, we are able to objectively characterize the dynamic evolution of neurophysiological activity induced by ketamine. By applying this tool to analyze neural data from both NHPs and humans, we obtain precise estimates of time-scales associated with neurophysiological phenomena observed in both species (e.g., alternating gamma slow-delta activities). Overall, our work provides methodological innovation to analyze switching spectral signatures as well as neuroscience inferences that result from applying this analytic tool to neural recordings from multiple NHPs and patients.

The paper is organized as follows. In the following section 3.2 we present our beta-HMM formulation and useful statistics based on the estimated beta-HMM. In section 3.3 we describe the electrophysiology datasets to which we apply our analysis framework. We present the results from our beta-HMM analyses in section 4. Finally, in section 5 we summarize the core contribution of our work, highlight the connections between our work and that of others, and suggest next steps.

## 3 Materials and methods

### 3.1 Ethics statement

All NHP procedures reported here followed the guidelines of the National Institutes of Health and were approved by the Massachusetts Institute of Technology’s Committee on Animal Care. The Partners Institutional Review Board (IRB) approved this human retrospective observational study. The IRB approved a waiver of consent for this study due to the anonymous nature of EEG data.

### 3.2 A hidden Markov model for beta distributed observations

#### 3.2.1 Reduced-order observations derived from multitaper spectrogram

We consider a sequence of scalar-valued single-channel voltage activity representing either LFP or EEG sampled at frequency *F*_*s*_ (in Hz). To quantify the spectral dynamics in the signal, we estimate time-varying spectra across a sequence of overlapping time-windows, each of duration Δ_*MT*_ = 1s, with 90% overlap between consecutive windows. The power spectral density (in dB) at the *n*-th time-window and corresponding to frequency *ω*_*i*_ (in Hz) is denoted by *S*_*n*_(*ω*_*i*_) (see glossary in Table A1 in S1 Appendix). We use the multitaper spectral estimation approach [54–56] (time-halfbandwidth product = 2, and number of tapers = 3) to calculate the sequence {*S*_*n*_(*ω*_*i*_)}, where *n* ∈ [1, *N*], on a high dimensional set of distinct frequencies {*ω*_*i*_: 0 < *ω*_*i*_ < *F*_*s*_/2}. We reduce the complexity of our subsequent analyses by summarizing the high-dimensional information *S*_*n*_({*ω*_*i*_}) by a low-dimensional vector ${\bar{S}}_{n}\left(\left\{\omega_{h}\right\}\right)$. This low-dimensional vector characterizes the instantaneous average power in *H* (= 7) distinct frequency bands between 0–50 Hz. We defined the frequency bands according to canonically described neural oscillation bands [53]: slow (0–1 Hz), delta (1–4 Hz), theta (4–8 Hz), alpha (8–12 Hz), beta (12–25 Hz), low gamma (25–35 Hz), and gamma (35–50 Hz) (see Sec A1 in S1 Appendix for additional detail). These bands encompass the frequencies considered in previous studies of ketamine-induced changes in EEG spectra in healthy human subjects and patients [10, 11] (see S9 Fig for an exploration of alternative frequency band specifications). The sequence ${\bar{S}}_{n}\left(\omega \right)$, where ***ω*** denotes the vector of frequency bands {*ω*_1_, …, *ω*_*H*_}, is the input to the beta-HMM analysis. (We use bold math symbols to indicate vector-valued quantities).

#### 3.2.2 Observation model

To facilitate the comparison of spectral observations across different data sets whose spectral power may vary in amplitude but represent comparable spectral profiles, we scale ${\bar{S}}_{n}\left(\omega \right)$ to new vector ***y***_*n*_ ∈ [0, 1]^*H*^ whose *h*-th element is calculated as,

$$\begin{array}{c} y_{nh}=\frac{1}{1+e^{-\lambda_{h}({\bar{S}}_{n}\left(\omega_{h}\right)-Q_{2}\left(\bar{S}\left(\omega_{h}\right)\right)}}\;\;\;\;,\text{for}\;h\in \left\{1,\cdots ,H\right\}\;\;, \end{array}$$ (1)

where $Q_{j}\left(\bar{S}\left(\omega_{h}\right)\right)$ denotes the function that operates on a real-valued sequence $\bar{S}\left(\omega_{h}\right)=\left\{{\bar{S}}_{n}\left(\omega_{h}\right)\right\}$, *n* ∈ [1, *N*] to yield the *j*-th quartile. The set of scaled power observations is denoted by ***Y*** = {***y***_*n*_}, *n* ∈ [1, *N*]. The user-defined parameter λ_*h*_ controls the mutual separation of points in the *y*_*nh*_-space relative to their corresponding map in the ${\bar{S}}_{n}\left(\omega_{h}\right)$-space. We set this value to be λ_*h*_ = 2log(3)/ $\left(Q_{3}\left(\bar{S}\left(\omega_{h}\right)\right)-Q_{1}\left(\bar{S}\left(\omega_{h}\right)\right)\right)$. The intuition behind the choice of parameters in the logistic function is that they result in scaling of the data such that the median is scaled to 0.5 and the data between the 1st and 3rd quartiles is scaled approximately linearly between [0.25, 0.75] (Sec A2 in S1 Appendix). Data below the 1st quartile and beyond the 3rd quartile are scaled non-linearly, such that very small values are scaled to be close to zero and very large values are scaled to be close to one. Compared to linear 0–1 scaling, the logistic scaling method is less sensitive to outliers which, in linear scaling, can lead to low variance observations and thus reduce the likelihood of detecting distinct spectral profiles. With logistic scaling, the data in $\bar{S}\left(\omega_{h}\right)$ is scaled such that the standard deviations of the observations in each frequency band (***y***_*h*_) are between [0.23, 0.31] for all data sets reported here.

In our model, we assume that for the *n*-th time-window, the observation ***y***_*n*_ is a manifestation of an underlying latent brain state, *z*_*n*_, at the same instant. We also assume that *z*_*n*_ is a random process that transitions among *K* possible discrete states, each with its own characteristic observation probability distribution whose mean corresponds to a distinct spectral profile. We represent a state-specific and frequency band-specific probability density function (pdf) of the observations as a standard beta distribution:

$$\begin{array}{c} p\left(y_{nh}|z_{n}=k;a_{hk},b_{hk}\right)=\frac{y_{nh}^{a_{hk}-1}{\left(1-y_{nh}\right)}^{b_{hk}-1}}{B(a_{hk},b_{hk})}\;\;. \end{array}$$ (2)

Here, *p*(*y*) denotes the pdf of a continuous-valued random variable *y*. In Eq (2), *a*_*hk*_ > 0 and *b*_*hk*_ > 0 are real-valued scalar parameters of the beta distribution that correspond to the scaled power in the *h*-th (*h* ∈ [1, *H*]) frequency band when the state *k* ∈ [1, *K*] is active. The normalization term $B(a_{hk},b_{hk})$ denotes the beta function [57].

Our assumption of statistical independence across the *H* distinct frequency bands conditioned on an underlying state, as implied in Eq (2), leads to the conditional joint pdf (jpdf) on ***y***_*n*_,

$$\begin{array}{c} p\left(y_{n}|z_{n}=k;\phi_{k}\right)=\prod_{h=1}^{H}p\left(y_{nh}|z_{n}=k;a_{hk},b_{hk}\right)\;. \end{array}$$ (3)

where ***ϕ***_*k*_ ≡ {*a*_*hk*_, *b*_*hk*_} corresponds to the set of 2*H* beta distribution parameters associated with state *k*. The set of beta parameters across all *K* states is denoted as ***ϕ*** ≡ {***ϕ***_*k*_} (Sec A3 in S1 Appendix). While the beta distribution can be multimodal when both *a*_*hk*_ and *b*_*hk*_ ≤ 1, we require that each state-specific pdf is unimodal. A multimodal pdf associated with a latent state would indicate that the same state is associated with two different spectral profiles, which will confound our intended interpretation of the states. Therefore, to ensure the unimodality in the marginal pdf’s we restrict the domain of the beta distribution parameters to avoid scenarios when both *a*_*hk*_ ≤ 1 and *b*_*hk*_ ≤ 1 for a given *h* and *k*. In summary, we chose to work with the beta distribution because it is a continuous probability distribution defined on the interval [0, 1]. Furthermore, it is part of the exponential family which leads to efficient maximum likelihood estimation (Sec A3 in S1 Appendix). The beta distribution is highly flexible as it allows for unimodal distributions where the modes can lie anywhere in [0, 1].

#### 3.2.3 State transition model

We assume the transition dynamics of the discrete-valued latent state process, *z*_*n*_, to be governed by first-order Markov transitions characterized by a constant *K* × *K* transition probability matrix, ***A*** = {*A*_*jk*_}, such that

$$\begin{array}{c} Pr\left(z_{n}=k|z_{n-1}=j\right)=A_{jk}\;\;\;,\;\text{where}\;\sum_{k=1}^{K}A_{jk}=1\;\;. \end{array}$$ (4)

Here, *Pr*(*z*) denotes the probability mass function of a discrete-valued random variable *z*. The initial state probability at the first time-window is characterized by a vector ***π*** = {*π*_*k*_} such that

$$\begin{array}{c} Pr\left(z_{1}=k\right)=\pi_{k}\;\;\;,\;\text{where}\;\sum_{k=1}^{K}\pi_{k}=1\;\;. \end{array}$$ (5)

Together, the observation model (Eq (3)), state transition matrix (Eq (4)), and initial state probabilities (Eq (5)) define our state-space model, which we refer to as the *beta-HMM*.

#### 3.2.4 Statistical analysis using beta-HMM parameters

The model parameters of the beta-HMM can be estimated from one or more independent EEG/LFP recording sessions (Sec A3 in S1 Appendix). The estimated beta-HMM provides reduced-order summaries of the scaled spectral dynamics. Between any two states, we can compare the spectral power in a given frequency band, as well as duration statistics derived from the transition matrix.

**Summarizing scaled spectral power in a given frequency band**. The set of beta distributions provides rich information about the scaled spectral observations from which the parameters are estimated. One helpful statistic to summarize each beta distribution is *Pr*(*Y*_*h*_ > 0.5|*Z* = *k*) where (*Y*_*h*_|*Z* = *k*)∼*Beta*(*a*_*hk*_, *b*_*hk*_). The notation (*Y*_*h*_|*Z* = *k*)∼*Beta*(*a*_*hk*_, *b*_*hk*_) denotes a random variable (*Y*_*h*_|*Z* = *k*) that is distributed according to the beta distribution *Beta*(*a*_*hk*_, *b*_*hk*_). A realization of (*Y*_*h*_|*Z* = *k*) would represent a scaled power observation in the *h*-th frequency band for a known latent state *Z* = *k*. *Pr*(*Y*_*h*_ > 0.5|*Z* = *k*) describes the probability that an observation from the *h*-th frequency band and *k*-th state is greater than the median scaled power in the *h*-th frequency band.

**Comparing scaled spectral power in a given frequency band between any two beta-HMM states**. One way to quantify how different any pair of beta distributions are from one another is to calculate the Kolmogorov-Smirnoff (KS)-distance from a large number of samples generated from the two distributions (we use 100,000 samples here). The KS-distance measures the maximum difference between two empirical CDFs, so values close to zero indicate that the underlying distributions are similar, and larger values indicate that the underlying distributions are dissimilar. Alternatively, we can leverage the analytic tractability of the beta distributions to analyze a difference random variable, Δ_*hjk*_ = *X*_*hj*_ − *X*_*hk*_, where *X*_*hj*_ ∼ *Beta*(*a*_*hj*_, *b*_*hj*_) and *X*_*hk*_ ∼ *Beta*(*a*_*hk*_, *b*_*hk*_). (In this section, we substitute (*Y*_*h*_|*Z* = *k*) with *X*_*hk*_ to simplify the subsequent notation.) Here, the parameter sets {*a*_*hj*_, *b*_*hj*_} and {*a*_*hk*_, *b*_*hk*_} respectively correspond to a state *j* and another state *k* in the *h*-th frequency band. We use *Pr*(Δ_*hjk*_ ≤ 0) to denote the cumulative distribution function (cdf) value at 0 for Δ_*hjk*_ ∈ [−1, 1] such that

$$\begin{array}{c} Pr\left(\Delta_{hjk}\leq 0;a_{hj},b_{hj},a_{hk},b_{hk}\right)=\int_{-1}^{0}p\left(\delta ;a_{hj},b_{hj},a_{hk},b_{hk}\right)d\delta \end{array}$$ (6)

where the pdf of the random variable Δ_*hjk*_ can be expressed at a given realization *δ* as,

$$\begin{array}{c} p\left(\delta ;a_{hj},b_{hj},a_{hk},b_{hk}\right)=\{\begin{array}{cc} \int_{0}^{1+\delta }p\left(x_{hj},\delta \right)dx_{hj}\; & -1<\delta \leq 0\\ \int_{\delta }^{1}p\left(x_{hj},\delta \right)dx_{hj} & 0<\delta <1 \end{array} \end{array}$$ (7)

where the joint pdf *p*(*x*_*hj*_, *δ*) is defined as,

$$\begin{array}{cc} p(x_{hj},\delta ) & =\frac{x_{hj}^{a_{hj}-1}{\left(1-x_{hj}\right)}^{b_{hj}-1}}{B(a_{hj},b_{hj})}\times \frac{{\left(x_{hj}-\delta \right)}^{a_{hk}-1}{\left(1-x_{hj}+\delta \right)}^{b_{hk}-1}}{B(a_{hk},b_{hk})}\;\;. \end{array}$$ (8)

A general approach to calculating probability distributions of difference random variables can be found in the work by Cook and Nadarajah [58]. In this case, if *Pr*(Δ_*hjk*_ ≤ 0) = *α*, then there is probability of *α* that *X*_*hj*_ ≤ *X*_*hk*_, or equivalently a probability of 1 − *α* that *X*_*hj*_ > *X*_*hk*_. In the ensuing discussion we adopt the following notational convention for clarity: For a within-subject pairwise comparison of HMM states estimated for a single NHP (Sec 4.3), we add a superscript indicating the NHP (e.g. $\Delta_{hjk}^{MJ}=X_{hj}^{MJ}-X_{hk}^{MJ}$, for NHP MJ). When we compare states between two NHPs, we add superscripts indicating both NHPs (e.g. $\Delta_{hjk}^{MJ,LM}=X_{hj}^{MJ}-X_{hk}^{LM}$, when comparing an HMM state from NHP MJ to one from NHP LM). When we perform pairwise comparison of HMM states estimated from the human OR dataset (Sec 4.4), we add the superscript H, $\Delta_{hjk}^{H}$.

**Comparing duration statistics between (i) any two beta-HMM states, and (ii) any two neurophysiological states each characterized by one or more beta-HMM states**. We present several statistics to describe the dynamics of the model. If *d*_*k*_ indicates a positive integer-valued random variable of the duration spent in state *k* after transitioning into it and before transitioning out to a different state, then the *mean duration* in that HMM state is given by

$$\begin{array}{c} {\hat{d}}_{k}=\sum_{d=1}^{\infty }d_{k}Pr\left(d_{k}\right)=\frac{1}{1-A_{kk}} \end{array}$$ (9)

where $Pr\left(d_{k}\right)\equiv Pr\left(z_{n:n+d_{k}}=k|z_{n}=k,z_{n-1}\neq k\right)=\left(1-A_{kk}\right)A_{kk}^{d_{k}-1}$, which holds for any *n* > 1 [35].

In cases where a subset of beta-HMM states, k* ⊂ {1, ⋯, *K*} corresponds to a neurophysiological phenomenon of interest (e.g. multiple HMM states corresponding to sub-states of gamma activity, or, multiple HMM states corresponding to the period between two consecutive gamma activities), we use the following Monte Carlo approach to estimate the consecutive time spent in this subset of beta-HMM states. Using the estimated transition matrix $\hat{A}$ and randomly sampled state *z*_1_, we generate a Markov sequence, *z*_1:*N*_ (*N* = 2000 in our study). For all *z*_*n*_’s taking values in k*, the corresponding state label is reassigned as *z*_*n*_ = 1. The states that do not take values in k* are reassigned values as *z*_*n*_ = 0. We then calculate the mean duration spent in the neurophysiological state, ${\hat{d}}_{k^{*}}$, as the mean number of consecutive time points where *z*_*n*_ = 1 holds. Similarly, for the same neurophysiological state ***k***^⋆^ we also calculate a *mean interval* statistic as the average number of consecutive timepoints where *z*_*n*_ = 0; this is an estimate of the time interval between the two consecutive occurrences of ***k***^⋆^. By repeating this procedure *N*_*MC*_ times (= 4000 in our study) we calculate median and 95% confidence intervals of the state-specific mean duration and mean interval statistics, together referred to as the duration statistics in the ensuing discussion.

### 3.3 Experimental recordings

#### 3.3.1 NHP LFP recordings under ketamine anesthesia

LFPs were recorded in multiple separate recording sessions from two rhesus macaques (*Macaca mulatta*) aged 14 years (NHP MJ, male, 13.0 kg) and 8 years (NHP LM, female, 6.6 kg). LFP recordings from 4 sessions in NHP MJ and 5 sessions in NHP LM were analyzed in this study. During each recording session, ketamine was administered as a single 20 mg/kg bolus intramuscular dose. This is a putative high dose for sedation and low dose for surgical anesthesia in NHPs [59–61]. Fifteen minutes prior to ketamine administration, glycopyrrolate (0.01 mg/kg) was delivered to reduce salivation and airway secretions. In both NHPs, LFPs were recorded from a 8 × 8 iridium-oxide contact microelectrode array (“Utah array”, MultiPort: 1.0 mm shank length, 400 *μ*m spacing, Blackrock Microsystems, Salt Lake City, UT) implanted in the frontal cortex (vlPFC). LFPs, recorded at 30 kHz, were low-pass filtered to 250 Hz and then downsampled to 1 kHz. LFPs were continuously recorded from 1–5 minutes prior to ketamine injection up to 18–20 minutes following ketamine injection. In the ensuing figures representing neural timeseries data, the time 0 denotes the time-point when the ketamine bolus was administered.

#### 3.3.2 Human EEG recordings under ketamine anesthesia

The data analyzed in this manuscript were acquired during an EEG study of ketamine-induced general anesthesia. We reviewed and selected 9 patients (Age: 51.8±11.7 years, Weight: 84.5±15.5 kg, mean ± standard deviation) from our database who were administered an intravenous bolus dose of ketamine as the sole hypnotic drug for the induction of general anesthesia. The ASA scores in all these 9 patients were less than or equal to 3, and none of the patients had a history of any neurological or psychiatric disorders. Prior to the ketamine bolus, patients were administered midazolam (n = 8; 1.81 ± 0.53 mg) and/or fentanyl (n = 7; 164.29 ± 80.18 mcg) for anxiolysis and to block the sympathetic response to laryngoscopy, respectively. To induce general anesthesia (GA) a bolus dose of ketamine (mean ± standard deviation; 182.22 ± 29.06 mg, 10 mg/ml) was administered, and intubation was carried out using succinylcholine, cisatracurium, or rocuronium for muscle relaxation. EEG data were acquired using Sedline monitor (Masimo Inc, USA). The standard Sedline Sedtrace electrode arrays were placed on the forehead that approximated the positions of Fp1, Fp2, F7, and F8, the ground electrode at Fpz, and the reference electrode 1 cm above Fpz. For our analysis, we use the data recorded from Fp2. Data were recorded with a pre-amplifier bandwidth of 0.5 to 92 Hz, a sampling rate of 250 Hz, with 16-bit, 29 nV resolution. Electrode impedance was less than 5 k*Ω* in each channel. We analyzed continuous EEG recordings starting from 2 minutes pre-ketamine bolus and up to 5.5 min—14 min post-ketamine bolus, and prior to the administration of any additional hypnotic drugs used to maintain GA.

## 4 Results

### 4.1 Testing the beta-HMM analysis framework against simulated ground truth

We first validated the ability of our beta-HMM analysis framework to accurately retrieve a known Markov sequence and associated observation distributions by simulating spectrograms generated from pre-specifed Markov processes (see Table A2 in S1 Appendix for the simulation algorithm). We simulated spectral dynamics comparable to those caused by ketamine by using a spectrogram ***S***({*ω*_*i*_}) calculated from one session of NHP experimental data (as described in Sec 3.2.1) as input to the algorithm. Additional inputs to this algorithm are user-prescribed HMM parameters, the number of states *K*, ***π*** and ***A***, as well as the duration *M* of the simulated data. This algorithm outputs a realization of the latent state path, ***z*** = {*z*_*m*_}, *m* ∈ [1, *M*], corresponding simulated spectrogram, ***S***^*sim*^({*ω*_*i*_}), and ***Y***. Salient features of the algorithm in Table A2 (S1 Appendix) are as follows. First, *K* distinct spectral clusters are created from the given NHP LFP spectrogram using an unsupervised clustering algorithm (different from beta-HMM) that does not respect the dependence across consecutive time-points. Then a Markov sequence is simulated using the known parameters, ***π*** and ***A***. Finally, to generate the spectrogram with *K* underlying latent states with Markov state transitions, a spectrum is selected for a given instant by uniformly sampling from one of the *K* spectral clusters that corresponds to the realization of the latent state at the same instant. As described in Sec 3.2, ***Y*** is calculated from ***S***^*sim*^({*ω*_*i*_}) and used to estimate a *K*-state beta-HMM (Sec A3 in S1 Appendix).

For a given *K*, we generate 100 realizations of ***Y*** based on *K* spectral clusters. For each realization of ***Y***, we estimate a *K*-state beta HMM to determine a set of estimated model parameters $\hat{\Theta }=\left\{\hat{\pi },\hat{A},\hat{\phi }\right\}$, and the estimated state path z* (calculated using the Viterbi algorithm). We assess goodness of fit for each realization of ***Y*** by comparing the estimated state path, z*, and model parameters, $\hat{\Theta }$, to the ground truth state path, ***z***, and model parameters, Θ, respectively.

To assess the accuracy of the estimated path z* compared to the known path ***z***, we calculated the fraction of time points for which the estimated and known path are identical:

$$\begin{array}{c} \text{Path}\;\text{Accuracy}=\frac{\sum_{n=1}^{N}I\left(z_{n},z_{n}^{*}\right)}{N},\;\text{where}\;I\left(z_{n},z_{n}^{*}\right)=\{\begin{array}{cc} 0 & z_{n}\neq z_{n}^{*}\\ 1 & z_{n}=z_{n}^{*} \end{array} \end{array}$$ (10)

To assess the accuracy of the observation distribution estimation, we calculated the mean KS-distance between the *HK* ground truth and estimated beta distributions. KS-distances close to zero indicate that the estimated and ground truth distributions are similar; the maximum possible KS-distance is 1. We also calculated the absolute errors in the estimated initial state and state transition probabilities, denoted respectively by *ϵ*_*A*_ and *ϵ*_*π*_,

$$\begin{array}{c} \epsilon_{A}=\frac{\sum_{j=1}^{K}\sum_{k=1}^{K}\left|A_{jk}-{\hat{A}}_{jk}\right|}{2K} \end{array}$$ (11)

$$\begin{array}{c} \epsilon_{\pi }=\frac{\sum_{k=1}^{K}\left|\pi_{k}-{\hat{\pi }}_{k}\right|}{2} \end{array}$$ (12)

**Fig 2:**
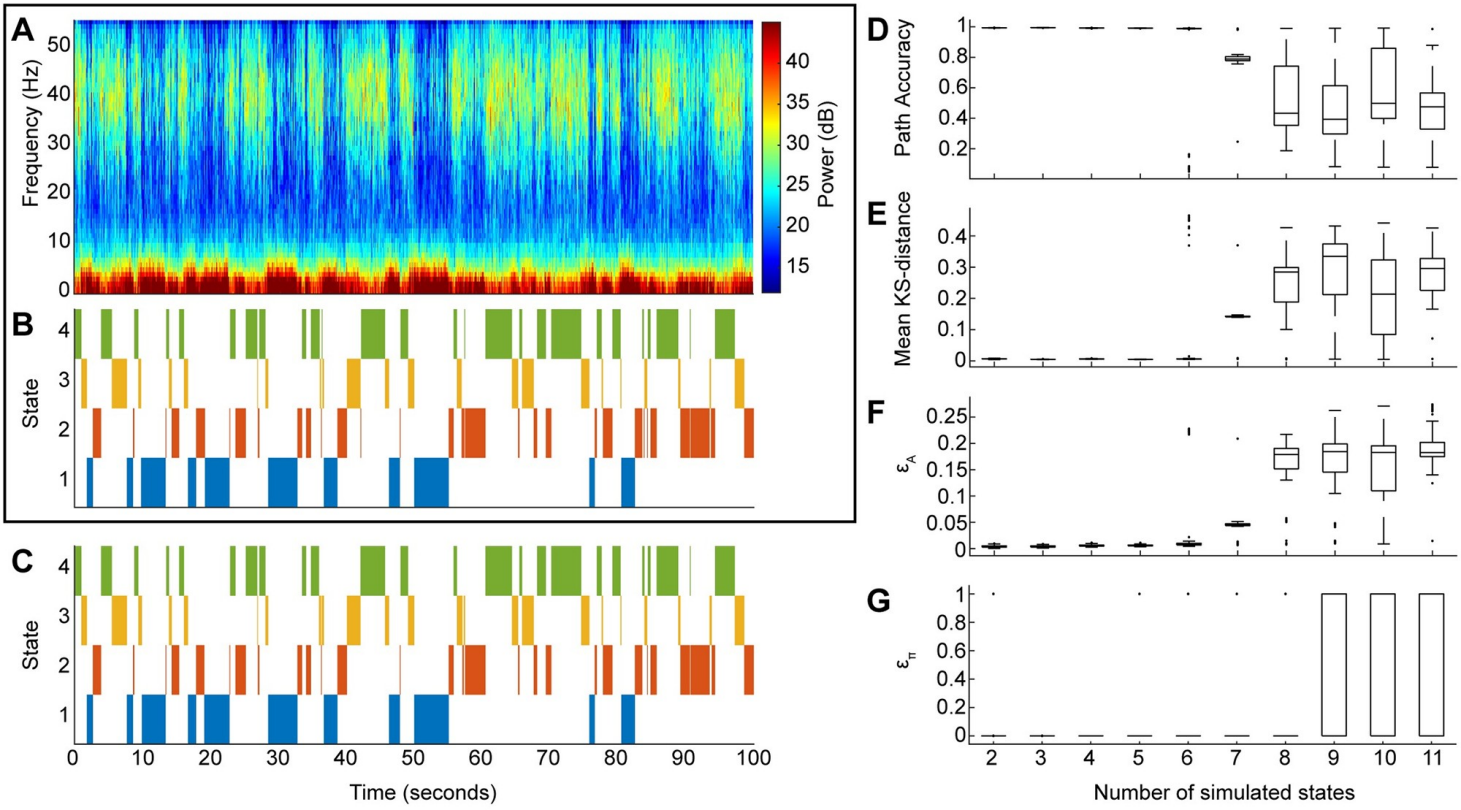
The beta-HMM tracks the dynamic structure of simulated spectrograms characterized by discrete state transitions (extracted from one sequence of ketamine-induced LFP in NHP MJ). (A) One realization of simulated spectrogram. (B) Markov path corresponding to the simulated spectrogram in panel A. (C) Optimal state trajectory estimated from simulated data in panel A via the beta-HMM analysis. (D-G) Goodness of fit analysis. Box-plots summarize the state path accuracy (Eq (10)) in panel D, the KS-distance between the estimated and the actual state-specific and frequency band-specific beta distributions (averaged across all states and all frequency bands) in panel E, the relative error in transition matrix (Eq (11)) in panel F, and relative error in the initial state probabilities (Eq (12)) in panel G. For each specified number of states, 100 realizations of spectrograms were simulated and the beta-HMM analysis was performed on each.

Note the the maximum possible value for $\sum_{j=1}^{K}\sum_{k=1}^{K}\left|A_{jk}-{\hat{A}}_{jk}\right|=2K$ and for $\sum_{k=1}^{K}\left|\pi_{k}-{\hat{\pi }}_{k}\right|=2$. Thus, the denominators scale the absolute errors to the range of [0, 1]. We report the median and 90% confidence intervals of each of these metrics calculated across 100 simulated spectrograms for each model order in Fig 2.

Simulation analysis revealed that the model parameters of simulated spectrograms, generated from ketamine-induced neural data and characterized by known Markov transition dynamics, can be accurately estimated with the beta-HMM framework for low (*K* < 7) model orders. The performance of the beta-HMM analysis framework on a typical simulated spectrogram (Fig 2A) demonstrates that the estimated state trajectory (Fig 2C) matches well with the actual state trajectory (Fig 2B). Across 400 simulated datasets for models with 2 through 5 states (100 simulations each), the estimated state trajectory was estimated with high accuracy (*PathAccuracy* > 0.98 for all of 400 independent simulations), the beta distributions were estimated with high accuracy (mean KS-distance <8.78 × 10^−3^), and the transition matrix was estimated with low relative error (*ϵ*_*A*_ < 0.01). For models with 2–5 states, the initial probability, which corresponds to a single data point, was also accurately estimated (*ϵ*_*π*_ < 3.09 × 10^−4^ for all but three of the 400 independent simulations). Beyond 6 states, there was a significant drop-off in estimation accuracy. A potential reason for this occurrence is redundancy across some of the *K* > 6 spectral clusters derived from the original NHP spectrogram, which may not contain more than 6 clusters of distinct neural activity. Overall, this numerical experiment generated detailed insights on the level of inaccuracy in our beta-HMMs when estimated from spectral data derived from real neural activity induced by ketamine. The simulations revealed that the beta-HMM framework was able to estimate Markov state trajectories and model parameters with high accuracy for models with 2–5 states.

### 4.2 Demonstration of the beta-HMM analysis on a single observation sequence of NHP LFP data (*L* = 1 case)

Using the numerically tested beta-HMM analysis framework, we analyzed a single session high-dose ketamine NHP LFP recording (Sec 3.3.1). From qualitative analysis of the time-series data and spectral estimates (S1 Fig), we identified two prominent neural signatures induced by ketamine: one is the gamma activity that has been previously described [10], and one is prominent slow-delta activity that also varies in power on a similar time scale to the gamma activity. The gamma and slow-delta activities tend to co-occur, resulting in four sub-states characterized by high or low gamma activity and high or low 0–4 Hz activity. We also sought to capture the transition from pre- to post- ketamine bolus neurophysiology, so we chose a model order of *K* = 5. We estimated a 5-state beta-HMM from a single sequence of LFP spectrogram.

**Fig 3:**
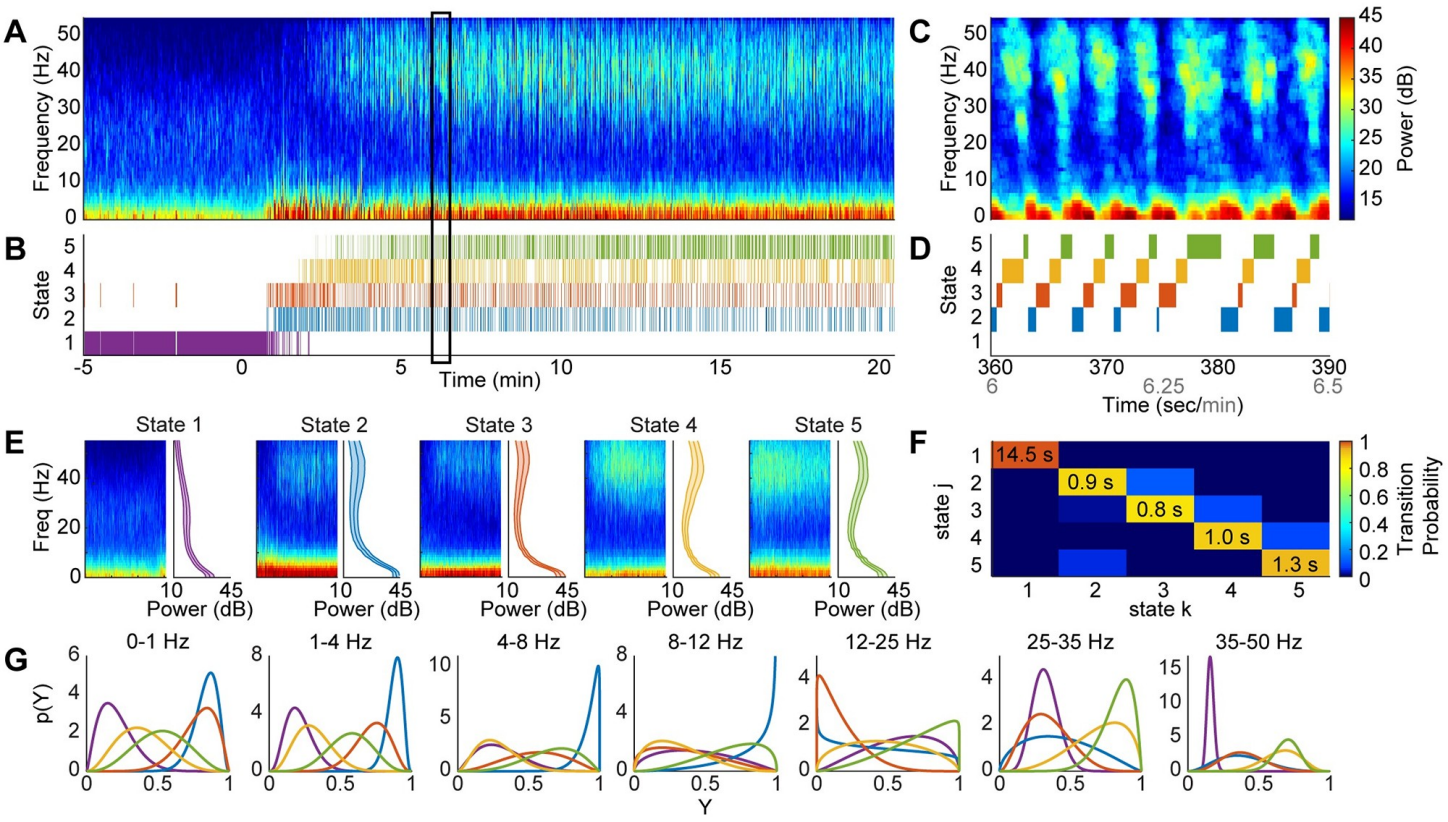
A 5-state beta-HMM estimates ketamine-induced neurophysiological dynamics from a single NHP LFP sequence. (A) Multitaper spectrogram of LFP where time 0 marks the time-point of administration of 20 mg/kg ketamine intramuscular bolus. (B) Estimated latent state trajectory. (C) Enlarged view of panel A for the epoch identified by the rectangular box. (D) Enlarged view of panel B for the epoch identified by the rectangular box. (E) Spectral clusters identified by the 5-state beta-HMM. Each spectral cluster has a characteristic signature as indicated by the mean spectrum and corresponding standard deviation. (F) The estimated transition matrix. The expected value of the duration spent in each state is indicated on the diagonal. (G) Frequency-band specific beta distributions (over scaled power (Y) in respective bands) estimated for each of the 5 states. Each color corresponds to a state, as denoted in panel B.

The 5-state beta-HMM provided a quantitative characterization of the neurophysiological dynamics induced by ketamine (Fig 3A and 3B). The beta-HMM analysis objectively identified alternating gamma slow-delta dynamics in NHPs similar to those described by Akeju, et al. [10]. We found that the HMM identified alternating states which corresponded to periods of prominent gamma oscillations and prominent slow-delta oscillations (Fig 3C and 3D). From visual inspection of the the state-segmented spectrograms and further analysis of beta distributions (Fig 3E and 3G and S4 Fig), we identified relevant neurophysiological states with distinct spectral signatures. For example, in the state-segmented spectrogram for state 5 (Fig 3E), we can see that high spectral power in the 25–35 Hz frequency band (*h* = 6) is associated with a high probability that (*Y*_6_|*Z* = 5)>0.5 (Fig 3G). (See Sec A6 in S1 Appendix for a tutorial-styled explanation of how the estimated beta pdf’s are used to analyze the spectral properties of the HMM states). Based on the beta distributions, we found that HMM states 4 and 5 both have high gamma power (25–35 Hz (*h* = 6): *Pr*(*Y*_6_ > 0.5|*Z* = 4) = 0.84, *Pr*(*Y*_6_ > 0.5|*Z* = 5) = 0.99; 35–50 Hz (*h* = 7): *Pr*(*Y*_7_ > 0.5|*Z* = 4) = 0.89, *Pr*(*Y*_7_ > 0.5|*Z* = 5) = 0.98). HMM states 2 and 3 have prominent slow-delta power (0–1 Hz (*h* = 1): *Pr*(*Y*_1_ > 0.5|*Z* = 2) = 1.00, *Pr*(*Y*_1_ > 0.5|*Z* = 3) = 0.97; 1–4 Hz (*h* = 2): *Pr*(*Y*_2_ > 0.5|*Z* = 2) = 1.00, *Pr*(*Y*_2_ > 0.5|*Z* = 3) = 0.96). Therefore, we consider HMM states 4 and 5 together to represent the neurophysiological gamma activity, and HMM states 2 and 3 to represent the slow-delta activity. In state 2, the low frequency activity extends into the theta (4–8 Hz, *h* = 3) and alpha (8–12 Hz, *h* = 4) ranges (*Pr*(*Y*_3_ > 0.5|*Z* = 2) = 1.00, *Pr*(*Y*_4_ > 0.5|*Z* = 2) = 0.93). The gamma and slow-delta activities tended to overlap (Fig 3C and 3D), resulting in moderate slow-delta power in state 5 (*Pr*(*Y*_1_ > 0.5|*Z* = 5) = 0.55, *Pr*(*Y*_2_ > 0.5|*Z* = 5) = 0.68).

The transition matrix (Fig 3F) quantifies the alternating dynamics illustrated in Fig 3C and 3D. From the transition matrix, we can also determine the expected duration of each state, as denoted on the diagonal terms indicating self-transition probabilities (Eq 9). From the transition probabilities and optimal state trajectory, we infer there is a high probability of the state sequence 2 → 3 → 4 → 5 → 2. This confirms the alternating dynamics between time-localized high power activity in the gamma and low frequency bands induced by ketamine. The beta-HMM provides further evidence that the neural activity induced by ketamine is distinct from pre-ketamine neural activity. States 2, 4, and 5 do not occur prior to the ketamine bolus (Fig 3A and 3B). Furthermore, HMM state 1 has sustained prevalence prior to the bolus and does not occur after a brief initial period (approximately 2 minutes) post-bolus. Therefore, HMM state 1 can be associated with a pre-ketamine neurophysiological state. This is further supported by the fact that the transition probability from any of the post-bolus states back to state 1 is less than 0.01.

### 4.3 Subject-specific beta-HMMs estimated from multiple recording sessions (*L* > 1 case) from 2 NHPs

A review of summary statistics from session-specific beta-HMMs estimated from multiple recording sessions of each NHP indicated the presence of subject-specific HMM states that have common features (both spectral and temporal) across sessions. This further suggested the utility of a subject-specific HMM for each NHP. Therefore, we fitted two 5-state beta-HMMs to multiple LFP recording sessions from 2 NHPs: 4 sessions for NHP MJ and 5 sessions for NHP LM (Sec 3.3.1). A key implementation feature in our beta-HMM analysis from multiple sessions (for a given group) is that we do not concatenate multiple sessions; rather we treat them as mutually independent in our EM algorithm implementation (as discussed in Sec A3 in S1 Appendix). Thus, the beta-HMM analysis respects the sequential nature of the observations within a session, while maintaining the distinction across multiple sessions. Since there were at least three days between each NHP session, we treated them as mutually independent and therefore utilized the EM algorithm based on Eq. (A4) in S1 Appendix to estimate the model parameters.

**Fig 4:**
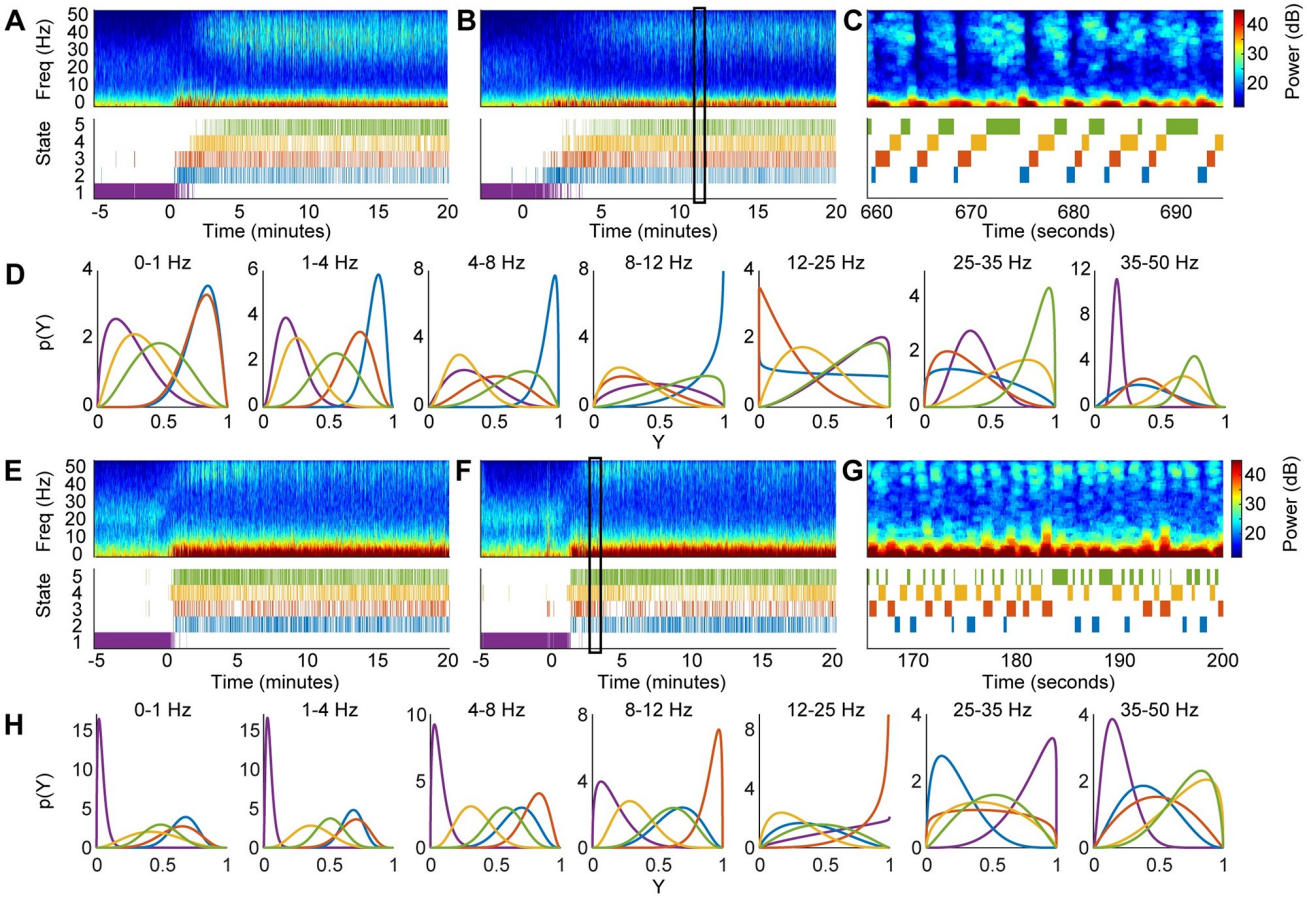
The 5-state beta-HMM can be fit across multiple sessions to create NHP-specific models. (A,B) Multitaper spectrograms of LFP and corresponding estimated latent state trajectories from 2 of 4 sessions in NHP MJ. (C) Enlarged view of a typical 35s epoch indicated by the rectangular portion in panel B. (D) Frequency band-specific beta pdfs for each of the 5 states estimated from the 4 sessions in NHP MJ (Y—scaled power). Each color corresponds to a state, as denoted in the state trajectories in panels A-C. (E,F) Multitaper spectrograms of LFP and corresponding estimated latent state trajectories from 2 of 5 sessions in NHP LM. (G) Enlarged view of a typical 35s epoch indicated by the rectangular portion in panel F. (H) Frequency band-specific beta pdfs for each of the 5 states estimated from the 5 sessions in NHP LM (Y—scaled power). Each color corresponds to a state, as denoted in the state trajectories in panels E-G.

We present the optimal state trajectories and state-specific and frequency band-specific observation distributions estimated from 4 of 9 total sessions of 2 NHPs in Fig 4 (see S2 Fig for all sessions). Although the amplitude of the spectral power tended to vary across sessions, our use of scaled power facilitated identification of common states with similar spectral profiles across multiple sessions (Fig 4A–4C and 4E–4G). The beta distributions provide detailed information about the spectral content of each HMM state, and we summarize the key results here. In both NHPs, we found that the beta distributions in each frequency band were unique across all states (KS-distance >0.04 for all pairwise comparisons; 140 comparisons = 10 pairs of states per frequency band per NHP × 7 frequency bands × 2 NHPs) (Fig 4D and 4H, S5 and S6 Figs). Consistent with the single session observations in NHP MJ, in both NHPs we found that HMM states 4 and 5 have high power in the 35–50 Hz (*h* = 7) range (NHP MJ: *Pr*(*Y*_7_ > 0.5|*Z* = 4) = 0.84, *Pr*(*Y*_7_ > 0.5|*Z* = 5) = 0.99; NHP LM: *Pr*(*Y*_7_ > 0.5|*Z* = 4) = 0.83, *Pr*(*Y*_7_ > 0.5|*Z* = 5) = 0.88). (See Sec A5 in S1 Appendix for a tutorial-styled explanation of how the estimated beta pdf’s are used to analyze the spectral properties of the HMM states.) In HMM states 4 and 5, we found that NHP LM had comparatively lower scaled power in the low gamma (25–35 Hz, *h* = 6) range (NHP MJ: *Pr*(*Y*_6_ > 0.5|*Z* = 4) = 0.75, *Pr*(*Y*_6_ > 0.5|*Z* = 5) = 0.99; NHP LM: *Pr*(*Y*_6_ > 0.5|*Z* = 4) = 0.44, *Pr*(*Y*_6_ > 0.5|*Z* = 5) = 0.53). In both NHPs, we found states 2 and 3 to have prominent slow-delta power (NHP MJ: 0–1 Hz (*h* = 1): *Pr*(*Y*_1_ > 0.5|*Z* = 2) = 0.98, *Pr*(*Y*_1_ > 0.5|*Z* = 3) = 0.97; 1–4 Hz (*h* = 2): *Pr*(*Y*_2_ > 0.5|*Z* = 2) = 1.00, *Pr*(*Y*_1_ > 0.5|*Z* = 3) = 0.95; NHP LM: 0–1 Hz (*h* = 1): *Pr*(*Y*_1_ > 0.5|*Z* = 2) = 0.95, *Pr*(*Y*_1_ > 0.5|*Z* = 3) = 0.82; 1–4 Hz (*h* = 2): *Pr*(*Y*_2_ > 0.5|*Z* = 2) = 0.98, *Pr*(*Y*_1_ > 0.5|*Z* = 3) = 0.95). Thus, in both NHPs we consider HMM states 4 and 5 together to represent the neurophysiological gamma activity, and states 2 and 3 to represent the slow-delta activity. As observed in the single session analysis, in NHP MJ state 2, the low frequency activity extends into the theta (4–8 Hz, *h* = 3) and alpha (8–12 Hz, *h* = 4) ranges (*Pr*(*Y*_3_ > 0.5|*Z* = 2) = 1.00, *Pr*(*Y*_4_ > 0.5|*Z* = 2) = 0.95). A similar result was observed in NHP LM state 3 (*Pr*(*Y*_3_ > 0.5|*Z* = 3) = 0.99, *Pr*(*Y*_4_ > 0.5|*Z* = 3) = 1.00). As demonstrated in Fig 3C and 3D, the multisession beta-HMM model captures the alternating dynamics induced by ketamine in both NHPs (Fig 4C and 4G). As noted in the single session analysis, there was some overlap between the gamma and slow-delta activity, resulting in moderate slow-delta power in state 5 (NHP MJ: *Pr*(*Y*_1_ > 0.5|*Z* = 5) = 0.46, *Pr*(*Y*_2_ > 0.5|*Z* = 5) = 0.62; NHP LM: *Pr*(*Y*_1_ > 0.5|*Z* = 5) = 0.48, *Pr*(*Y*_1_ > 0.5|*Z* = 5) = 0.55).

**Fig 5:**
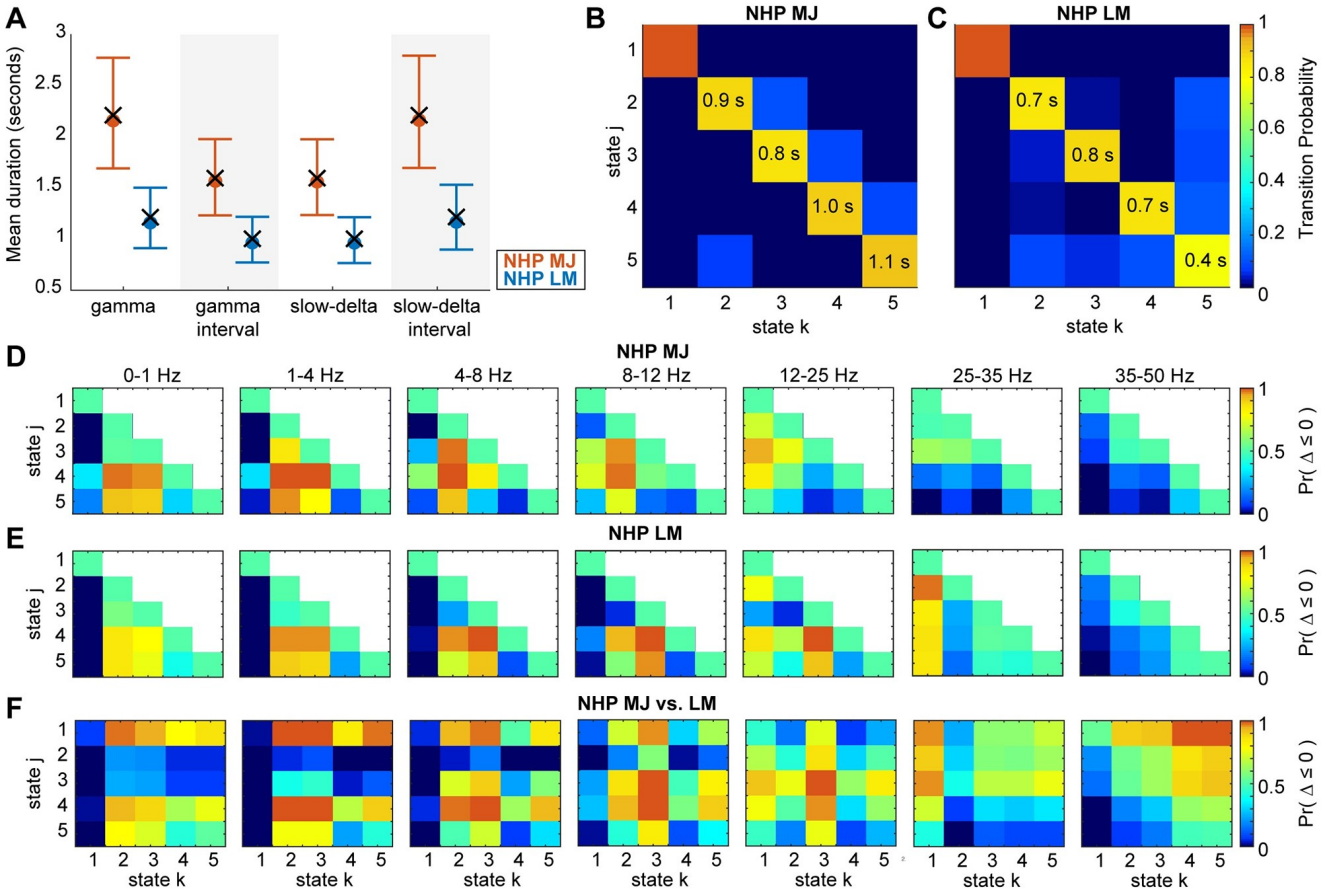
The estimated beta pdfs and transition matrices characterize the underlying dynamics of NHP LFP data following a 20 mg/kg bolus of ketamine. (A) Duration statistics corresponding to gamma and slow-delta activities in NHP MJ and NHP LM. For each subject, 4000 Markov sequences of length *N* = 2000 were simulated using the subject-specific estimated transition matrix. The mean duration and interval corresponding to the gamma and slow-delta activities were calculated for each realization of these sequences. Median and 95% confidence bounds across all the subject-specific simulated sequences are indicated in orange (NHP MJ) or blue (NHP LM). The mean duration and interval calculated from the estimated state trajectory (output of the Viterbi algorithm which uses the maximum likelihood beta-HMM parameters and the observations as input) are indicated by a cross (×) symbol. (B,C) The transition matrices for NHP MJ and NHP LM with expected state duration (in seconds) indicated on the diagonal for the states that occur following the ketamine bolus. (D-F) Heatmaps indicating the probability of the event that scaled power in state *j* (of NHP MJ in panels D and F, and of NHP LM in panel E) is less than or equal to the scaled power in state *k* (of NHP MJ in panel D, and of NHP LM in panels E and F) for each of the 7 frequency bands. Note that in panels D and E, the upper triangle (1 < *j* < *k* < 5), omitted to avoid redundancy, is equal to 1 minus the lower triangle (1 < *k* < *j* < 5). The color indicates *Pr*(Δ ≤ 0) which represents *Pr*(*X*_*hj*_ − *X*_*hk*_ ≤ 0), where *X*_*hj*_ is a random variable characterized by state *j*’s beta pdf in frequency band *h* and *X*_*hk*_ is a random variable characterized by state *k*’s beta pdf in the same frequency band *h*.

We used the subject-specific HMMs to further analyze the duration statistics and spectral content across states, both within a subject as well as between the two subjects (Fig 5). We found significant differences in the neurophysiological state dynamics between the two NHPs (Fig 5A–5C). The mean duration of the gamma activity, ${\hat{d}}_{4,5}$ (i.e. consecutive time-windows spent in states 4 or 5), was 2.2*s*([1.7, 2.8]*s*) in NHP MJ, and 1.2*s*([0.9, 1.5]*s*) in NHP LM. The mean duration of the slow-delta activity, ${\hat{d}}_{2,3}$, was 1.6*s*([1.2, 2.0]*s*) in NHP MJ, and 1.0*s*([0.8, 1.2]*s*) in NHP LM. Since the states dominated by gamma or slow-delta activities in NHPs alternate, the average interval between two consecutive periods of high gamma power is equal to the average duration of the slow-delta oscillations, and vice versa. The durations of both the gamma and slow-delta activities were longer in NHP MJ. From the transition matrices (Fig 5B and 5C), it is also apparent that the expected duration in each HMM state in NHP MJ is greater than that of NHP LM, indicating faster state transition dynamics in the latter subject.

We used the ML estimated beta distributions to perform pairwise comparisons of the scaled power between any two states from either NHP (Fig 5D and 5E). By investigating *Pr*(Δ_*hjk*_ ≤ 0;*a*_*hj*_, *b*_*hj*_, *a*_*hk*_, *b*_*hk*_) (per Sec 3.2.4), we were able to determine the probability that HMM state *j* had lower scaled power than HMM state *k* in the *h*-th frequency band. For this section, we simplify the notation by referring to *Pr*(Δ_*hjk*_ ≤ 0;*a*_*hj*_, *b*_*hj*_, *a*_*hk*_, *b*_*hk*_) as *Pr*(Δ_*hjk*_ ≤ 0), and summarize the key findings. In both NHPs, we found that the scaled powers in the 0–1 Hz and 35–50 Hz bands during the pre-ketamine state (HMM state 1) were lower relative to every other state ($0.00<Pr(\Delta_{hj,1}^{MJ}\leq 0)<0.34$ and $0.00<Pr(\Delta_{hj,1}^{LM}\leq 0)<0.16$ for *h* ∈ {1, 7} and *j* ∈ [2, *K*]). This provides further evidence that the oscillatory dynamics in these two frequency bands were most significantly altered by ketamine. NHP MJ had lower scaled power in the 25–35 and 35–50 Hz ranges in states 2 and 3 compared to states 4 and 5 ($0.01<Pr(\Delta_{hjk}^{MJ}\leq 0)<0.24$ for *h* ∈ {6, 7}, *j* ∈ {4, 5}, and *k* ∈ {2, 3}). This is consistent with the observation that the gamma activity (represented by states 4 and 5) in NHP MJ occurred broadly between 25–50 Hz. NHP LM also had lower scaled power in the 35–50 Hz range in states 2 and 3 compared to states 4 and 5 ($0.13<Pr(\Delta_{5,j,k}^{LM}\leq 0)<0.25$ for *j* ∈ {4, 5}, and *k* ∈ {2, 3}). However, state 4 and 5 in NHP LM had similar 25–35 Hz scaled power to state 3 ($0.45<Pr(\Delta_{6,j,3}^{LM}\leq 0)<0.52$ for *j* ∈ {4, 5}). This is consistent with the observation that the gamma activity (again represented by states 4 and 5) occurred primarily between 35–50 Hz in NHP LM. In both NHPs, states 2 and 3 had higher scaled power in the 0–1 and 1–4 Hz ranges compared to states 4 and 5 ($0.79<Pr(\Delta_{hjk}^{MJ}\leq 0)<1.00$ and $0.76<Pr(\Delta_{hjk}^{LM}\leq 0)<0.97$ for *h* ∈ {1, 2}, *j* ∈ {4, 5}, and *k* ∈ {2, 3}).

We further leveraged the subject-specific beta-HMMs to compare beta distributions between any HMM state in NHP MJ and any HMM state in NHP LM (Sec 3.2.4, Fig 5F). In the 35–50 Hz range, we found that HMM states 4 and 5 in NHP MJ were most similar to the HMM states 4 and 5 in NHP LM ($0.47<Pr(\Delta_{7,jk}^{MJ,LM}\leq 0)<0.65$ for *j* ∈ {4, 5} and *k* ∈ {4, 5}). Likewise, in the 35–50 Hz range, we found states 2 and 3 in NHP MJ were most similar to states 2 and 3 in NHP LM ($0.53<Pr(\Delta_{7,jk}^{MJ,LM}\leq 0)<0.64$ for *j* ∈ {2, 3} and *k* ∈ {2, 3}). NHP LM had more dramatic ketamine-induced increases in the slow-theta frequency ranges than NHP MJ, resulting in lower relative power in state 1 in NHP LM compared to any state in NHP MJ ($0.00<Pr(\Delta_{h,j,1}^{MJ,LM}\leq 0)<0.07$ for *h* ∈ [1, 3] and *j* ∈ [1, 5]).

### 4.4 Human-specific beta-HMM estimated using observations from multiple patient subjects (*L* > 1 case)

**Fig 6:**
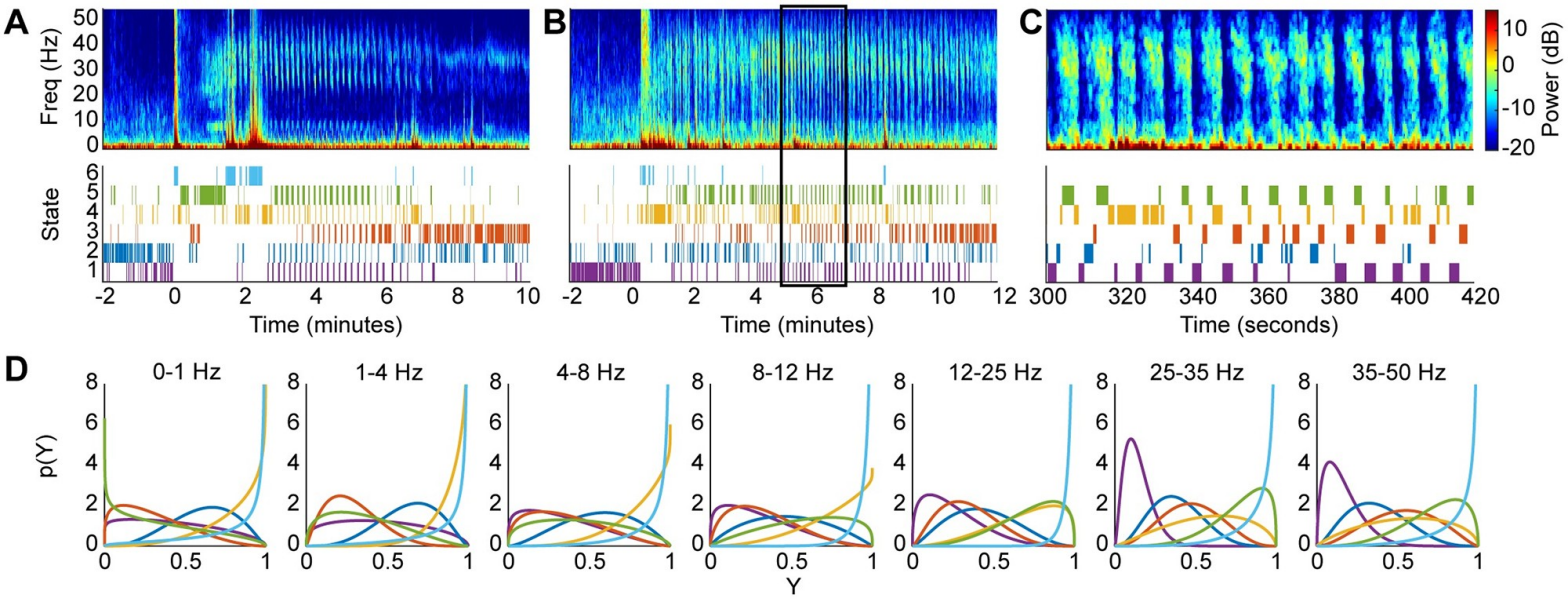
The 6-state beta-HMM can be fit across independent EEG recordings from multiple human subjects to create a typical human-specific model. (A-B) Multitaper spectrograms of EEG and corresponding estimated latent state trajectories from 2 of the 9 human subjects. (C) Enlarged view of a typical 120s epoch indicated by the rectangular portion in panel B. (D) Frequency band-specific beta pdfs for each of the 6 states estimated from the 9 EEG sessions (Y—scaled power). Each color corresponds to a state, as denoted in the state trajectories in panels A-C.

We investigated if the beta-HMM analysis framework could be applied to infer the common ketamine-induced neurophysiological state dynamics observed in the scalp EEG of multiple OR patients (Sec 3.3.2). We fit a single beta-HMM to independently collected EEG data from *L* = 9 human patients and generated a quantitative description of ketamine-induced neurophysiological dynamics for a typical patient (Fig 6). For this analysis, we chose a model order of *K* = 6 states, which allowed for classification of broadband high power, typical of noise artifacts in the OR, as an additional HMM state.

As with NHPs, we found that the beta distributions in each frequency band were unique across all states (KS-distance >0.04 for all pairwise comparisons; 105 comparisons = 15 pairs of states per frequency band × 7 frequency bands) (Fig 6D and S7 Fig). While there were differences in the ketamine-induced spectrograms between human patients (S3 Fig), the state-specific spectral signatures characterized by the beta distributions were overall consistent across all 9 patients (S8 Fig). We again found that HMM states 4 and 5 had high scaled power in the low gamma (25–35 Hz, *h* = 6) frequency range (*Pr*(*Y*_6_ > 0.5|*Z* = 4) = 0.62, *Pr*(*Y*_6_ > 0.5|*Z* = 5) = 0.93). In state 5, the high scaled power extended to the gamma (35–50 Hz, *h* = 7) range (*Pr*(*Y*_7_ > 0.5|*Z* = 5) = 0.87). Thus, states 4 and 5 also represent the neurophysiological gamma activity in human EEG. States 2 and 4 had dominant slow-delta power (0–1 Hz (*h* = 1): *Pr*(*Y*_1_ > 0.5|*Z* = 2) = 0.71, *Pr*(*Y*_1_ > 0.5|*Z* = 4) = 0.93; 1–4 Hz (*h* = 2): *Pr*(*Y*_2_ > 0.5|*Z* = 2) = 0.77, *Pr*(*Y*_1_ > 0.5|*Z* = 4) = 0.99), and thus characterize ketamine-induced slow-delta activity. In State 4, the low frequency activity extended into the the theta (4–8 Hz, *h* = 3) and alpha (8–12 Hz, *h* = 4) ranges (*Pr*(*Y*_3_ > 0.5|*Z* = 4) = 0.94, *Pr*(*Y*_4_ > 0.5|*Z* = 4) = 0.88).

There were distinct neurophysiological differences between the human EEG and the NHP LFP (Sec 4.3). First, the gamma activity in humans (captured by states 4 and 5) was lower in frequency and extended into the beta (12–25 Hz, *h* = 5) frequency range (*Pr*(*Y*_5_ > 0.5|*Z* = 4) = 0.81, *Pr*(*Y*_5_ > 0.5|*Z* = 5) = 0.86). Second, in the human EEG, state 4 had both prominent gamma and slow-delta activity, indicating that the gamma and slow-delta activities overlapped rather than alternated. Third, there was high slow-delta power and, in some patients, high gamma power (S3 Fig) throughout the human EEG recordings, which resulted in HMM states that were present both before and after the ketamine bolus. Finally, soon after the time of the ketamine bolus, in several patients (Fig 6B and S3 Fig), there was broadband high power that, in the context of OR cases, is difficult to distinguish from noise. This activity is classified with the 6-th state, which also corresponds to periods of obvious noise artifacts (e.g. at t = 0 min in Fig 6A).

**Fig 7:**
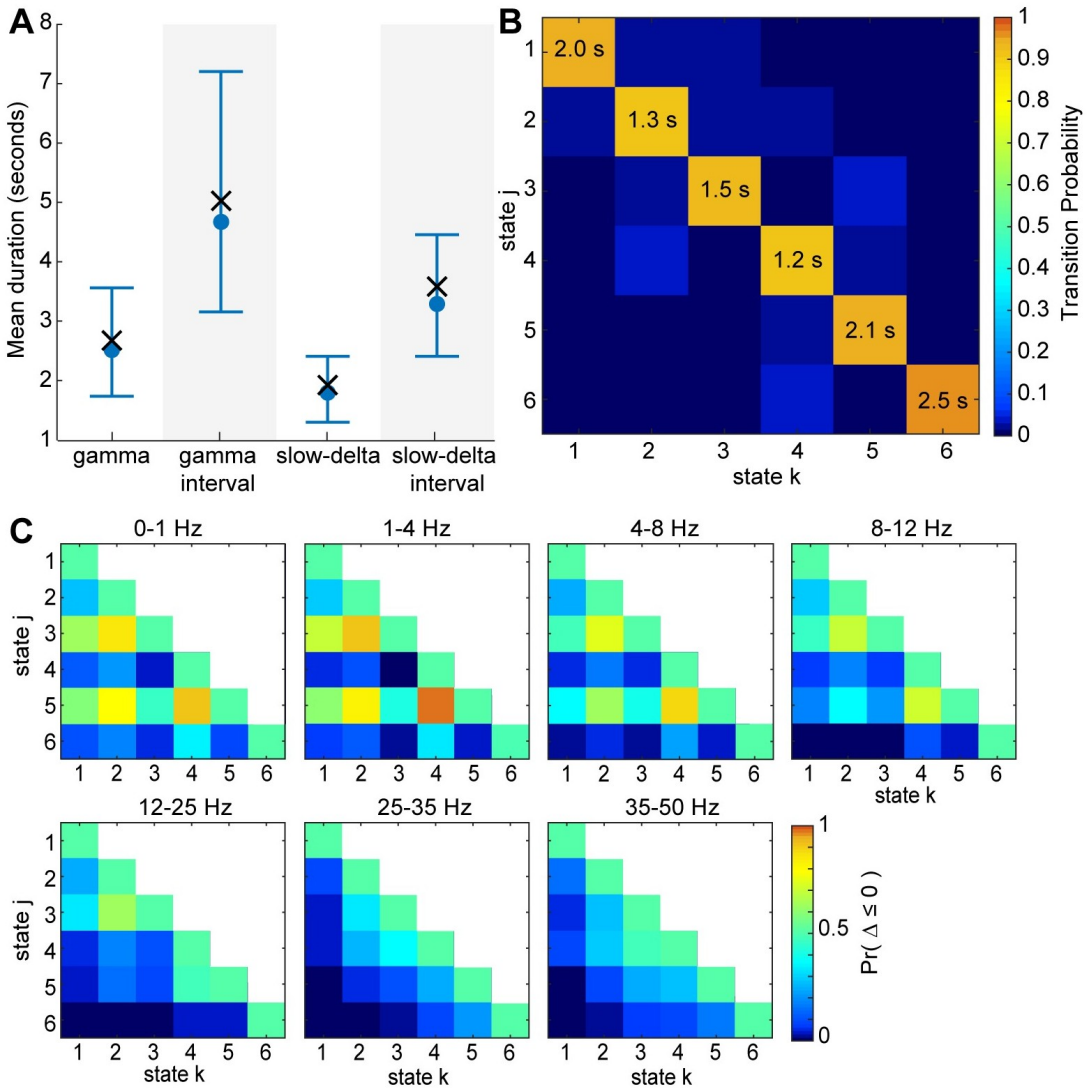
The estimated beta pdfs and transition matrix characterize the underlying dynamics of human scalp EEG under ketamine-induced general anesthesia. (A) Duration statistics corresponding to gamma and slow-delta activities. 4000 Markov sequences of length *N* = 2000 were simulated using the estimated transition matrix. The mean duration and interval corresponding to the gamma and slow-delta activities were calculated for each realization of these sequences. Median and 95% confidence bounds across all the simulated sequences are indicated in blue. The mean duration and interval calculated from the estimated state trajectory (output of the Viterbi algorithm which uses the maximum likelihood beta-HMM parameters and the observations as input) are indicated by a cross (×) symbol. (B) The transition matrices for NHP MJ and NHP LM with expected state duration (in seconds) indicated on the diagonal for all states. (C) Heatmaps indicating probability of the event that scaled power in state *j* is less than or equal to the scaled power in state *k* for 1 ≤ *k* ≤ *j* ≤ 6 and for each of the 5 frequency bands. Note that the upper triangle (1 < *j* < *k* < 6), omitted to avoid redundancy, is equal to 1 minus the lower triangle (1 < *k* < *j* < 5). The color indicates *Pr*(Δ ≤ 0) which represents *Pr*(*X*_*hj*_ − *X*_*hk*_ ≤ 0), where *X*_*hj*_ is a random variable characterized by state *j*’s beta pdf in frequency band *h* and *X*_*hk*_ is a random variable characterized by state *k*’s beta pdf in the same frequency band *h*.

Quantitative analysis of the beta-HMM dynamics (Fig 7A) revealed that the mean duration of the gamma activity, ${\hat{d}}_{4,5}$, was 2.5*s*([1.7, 3.6]*s*). The mean interval between consecutive occurrences of the gamma activity, ${\hat{d}}_{1,2,3}$, was 4.7*s*([3.1, 7.2]*s*). The mean duration of the slow-delta activity ${\hat{d}}_{2,4}$ was 1.8*s*([1.3, 2.4]*s*). The mean interval between consecutive occurrences of slow-delta activity, ${\hat{d}}_{1,3,5}$, was 3.2*s*([2.4, 4.5]*s*). Furthermore, the human EEG also tends to transition cyclically through the HMM states induced by ketamine (Fig 7B)—the most probable transition sequence, starting from state 1, is 1 → 2 → 3 → 5 → 4 → 2. All states have a low probability of transitioning to state 6 (*A*_*j*,6_ < 0.012 for *j* ∈ [1, 5]), further indicating that this state captures spurious noise artifacts.

Further quantitative analysis (Fig 7C) of human EEG beta-HMM parameters revealed higher scaled power in the 25–35 Hz range for HMM states 4 and 5 compared to states 1–3 ($0.00<Pr(\Delta_{6,j,k}^{H}\leq 0)<0.37$ for *j* ∈ {4, 5} and *k* ∈ [1, 3]). For state 5, the increased power extended into the 35–50 Hz range ($0.01<Pr(\Delta_{6,5,k}^{H}\leq 0)<0.24$ for and *k* ∈ [1, 3]). States 2 and 4 had the highest power between 0–4 Hz ($0.78<Pr(\Delta_{hjk}^{H}\leq 0)<1.00$ for *h* ∈ {1, 2}, *j* ∈ {2, 4}, and *k* ∈ {1, 3, 5}). As previously noted, there was a strong slow oscillation throughout the human recordings, so the ketamine-induced changes in the 0–1 Hz frequency range were more subtle than in the NHPs. Finally, through this analysis, it is again clear that state 6 was characterized by broadband high power ($0.00<Pr(\Delta_{h,6,k}^{H}\leq 0)<0.35$ for *h* ∈ [1, 7] and *k* ∈ [1, 5]).

## 5 Discussion

Our beta-HMM analysis framework provided parsimonious summaries of ketamine-induced slow-gamma alternating dynamics for NHP LFP and human EEG recordings following high-dose ketamine administration. The input to the analysis framework was one or more sequences of instantaneous power in multiple canonical frequency bands. The power values were averaged across frequencies within each discrete band and scaled between 0 and 1. The scaling enabled identification of latent HMM states each with a distinct probability distribution on power values lying between a very low (corresponding to a 0) and a very high value (corresponding to a 1) in each of the chosen frequency bands, irrespective of the absolute value of spectral estimates. A key assumption incorporated in the HMM is that at any instant in time, the probability distribution from which the observation is sampled depends only on the discrete value of an underlying latent state process at the same instant. This state process itself is assumed to evolve according to a first-order Markovian transition dynamics. The key outputs of our analysis comprised the model parameters estimated using an EM algorithm and a corresponding state trajectory estimated using the Viterbi algorithm. The state trajectory provided an objective and efficient segmentation of LFP and EEG data. The estimated state-specific and frequency band-specific beta pdf’s characterized the spectral representation of each HMM state, whereas the transition matrix characterized the underlying dynamics. Using the estimated observation pdf’s, we objectively defined neurophysiological activities (e.g. gamma activity, slow-delta activity) in terms of underlying HMM states. Furthermore, by using the estimated state transition matrices, we calculated mean duration and mean interval statistics (as defined in Sec 3.2.4) corresponding to each of these neurophysiological activities.

Our analyses revealed an alternating pattern of states characterized primarily by gamma and slow-delta activities. The mean duration of the gamma activity was 2.2s ([1.7, 2.8]s) and 1.2s ([0.9, 1.5]s) for the two NHPs, and 2.5s ([1.7, 3.6]s) for the nine human subjects. The mean duration of the slow-delta activity was 1.6s ([1.2, 2.0]s) and 1.0s ([0.8, 1,2]s) for the two NHPs, and 1.8s ([1.3, 2.4]s) for the nine human subjects. Our characterizations of the alternating gamma slow-delta activity revealed five sub-states that show regular sequential transitions. Thus, our objective beta-HMM analysis framework enabled us to precisely characterize the dynamics of ketamine-induced gamma and slow-delta activities, and to compare the time-scales of these neurophysiological states between subjects of the same species.

Our work advances the development and application of LFP or EEG data analysis tools. The beta-HMM analysis framework falls under the broad category of research aimed at developing time-frequency state space models to analyze neural dynamics [43, 52, 62–65], and applying them in principled interpretation of neural time-series data for unconsciousness research [11, 62]. A few key distinguishing features of the beta-HMM analysis framework are as follows. The assumption of state-specific and frequency-band specific beta distributions as marginal distributions of the instantaneous observation vectors allows for a flexible framework to characterize highly variable spectral profiles. This assumption, along with the assumption of Markovian state transitions, allowed us to directly characterize the discontinuous switching among multiple spectral profiles, as well as identify segments with similar spectral properties (Figs 3C, 3D; 4C, 4G; and 6C). Furthermore, our beta-HMM analysis framework accounts for the disjoint nature of data from separate sessions without requiring any concatenation of sessions or consequently any arbitrary choice of concatenation order. Potential extensions of this work on the statistical methods development front may include modeling the transition matrix as time-varying, which would account for longer time-scale variations (e.g. varying drug-levels). Furthermore, generalizing the current single-channel LFP or EEG data-based analysis framework to incorporate multi-channel LFP or EEG data would allow for neurophysiological characterization of ketamine-induced altered arousal states based on spatio-temporal statistical relationships in the observed data. Going beyond the context of ketamine-induced altered arousal states, the analysis framework itself can be relevant in general use-cases where LFP or EEG data demonstrate discontinuous, repeating transitions among time-limited, band-limited spectral signatures, such as those observed during sleep [43, 66] or burst suppression in general anesthesia [67, 68].

Our beta-HMM research can inform future neuroscience inquiries. While there are several hypotheses for the generation of gamma activity under ketamine [21], there is not yet a clear understanding of how the alternating gamma slow-delta activity is generated. Preliminary work indicates that there may be a cycle of cortical inhibition and dis-inhibition due to activity-dependent ketamine NMDAR inhibition of cortical interneurons and pyramidal neurons [22]. Our analyses of the NHP datasets using the beta-HMM led to the identification of neurophysiological activities that were primarily associated with the period following administration of a ketamine bolus. The summary statistics from each NHP (Fig 5) inform *which* frequency bands show significant activity, and *how* this activity varies over time. The duration and interval statistics of the alternating gamma and slow-delta activities that we estimated for NHP LFP can serve as quantitative constraints in the design of rhythm-generating neuronal microcircuit models that would mimic the neurophysiological dynamics caused by ketamine, similar to ones previously done for other anesthetics [23, 24, 26, 69, 70]. Furthermore, since ketamine is also used in treatment of depression [71, 72], in pharmacologic models for schizophrenia [20, 73, 74], and in studies of altered states of consciousness [75], insights into ketamine’s effects on brain state dynamics will provide neuroscientific insight beyond the field of anesthesia.

Our beta-HMM research can also inform future clinical inquiries. Ketamine is known to produce distinct oscillatory signatures in the electroencephalogram (EEG) of healthy volunteers and patients [9, 10]. These oscillatory signatures are starkly different from those produced during propofol-induced unconsciousness [76, 77]. These differences in oscillatory dynamics, particularly the presence of alternating gamma and slow-delta activities under ketamine, indicate that spectral markers of propofol-induced unconsciousness are not reliable markers for tracking ketamine-induced altered arousal states. However, in both the NHP LFP and human EEG recordings, we observed dramatic changes in the oscillatory state dynamics between the awake and post-ketamine states, suggesting that the alternating gamma slow-delta phenomena induced by ketamine is a marker of altered arousal. The existence of consistent neurophysiological activity observed in EEG following ketamine bolus administration in multiple patients indicates promise in precise clinical monitoring of these states. Towards this goal, as demonstrated in this work, the beta-HMM analysis framework can provide precise, objective characterization of the neurophysiological dynamics associated with ketamine-induced altered states of arousal. In summary, our work can be regarded as a part of the ongoing neuroscience research efforts to investigate alternating dynamical states of the brain and how these states transition from one to the other [78]. Alternating brain states have been previously identified in anesthesia-induced altered states of consciousness across different imaging modalities, species, analytic tools and anesthetic drugs [11, 79, 80]. The experimental and analytic framework developed here can be used to investigate differences in brain state dynamics between awake and altered states of consciousness. This investigation could be relevant in future studies looking into the theories of consciousness (e.g. [81]) as well as to improve the understanding of mechanism of actions of these drugs in therapeutic applications (e.g. [13, 14]).

There are two primary limitations in both the data set that we analysed here and in our assumptions. The first limitation is a need for prospective studies in humans and animals with carefully recorded simultaneous neural, behavioral response and physiological activities. Future experimental studies with larger cohorts can address the current study’s limitation due to low number of subjects in both the NHP LFP and human EEG analyses. The consistency of the alternating gamma-slow spectral activity from 9 patients and 2 NHPs during ketamine-induced altered arousal states indicate that transition dynamics between the gamma and slow-delta neurophysiological states can be a candidate biomarker of ketamine-induced unconsciousness. However, to reliably establish these EEG or LFP correlates of unconsciousness, experimental studies in healthy volunteers and animal subjects with simultaneous monitoring of behavioral response, physiological parameters and neural activity will be essential. The second limitation is a need for extension to real-time tracking of neurophysiological states during ketamine-induced general anesthesia. Our current approach utilizes the entire data set to calculate appropriate scaling factors for transforming spectral power to real numbers lying between 0 and 1, and thus (in the present formulation) cannot be executed in real time. A larger study may allow for the development of a generalized scaling approach. This would facilitate objective identification of ketamine neurophysiological states in real-time. While a common limitation of EEG analysis in real-time OR settings is the presence of motion and other noise artifacts in recordings, the beta-HMM framework is well poised to handle these artifacts, as observations that fall far outside the distribution of the signal can be assigned to a distinct HMM state.

In conclusion, we have developed a detailed analysis framework for ketamine-induced neurophysiological phenomena. Our work provides a methodological innovation to analyze switching spectral dynamics in single-channel electrophysiological data. The scaling of the frequency band-wise power to [0, 1] interval followed by its analyses in the beta-HMM framework facilitates estimation across multiple recording sessions, as well as comparisons between sessions. To our knowledge, this is the first application of an HMM with beta observation distributions for characterizing neural data. Our work also provides insights into the neural dynamics due to ketamine anesthesia. By applying our methodological innovation to LFP data from NHP subjects and EEG data from human patients collected during ketamine anesthesia, we have identified distinct neurophysiological states by their spectral signatures and their duration statistics. Our quantitative findings will inform future neurophysiological models as well as clinical biomarker search. Also, the generalizability of the beta-HMM framework indicates utility beyond what has been reported here. Future work will investigate how the spectral dynamics revealed by our analysis contribute to ketamine-induced altered states of arousal.

## Supporting information

S1 Appendix In the appendix, we provide additional detail in the calculation of band-wise power (Sec A1), scaling of the beta-HMM observations (Sec A2), estimation of model parameters (Sec A3), and EM algorithm (Sec A4). We also provide the algorithm for simulating a spectrogram with known Markov model parameters (Sec A5, Table A2), and a tutorial on interpreting beta distributions (Sec A6). In Table A1, we provide a glossary of mathematical symbols.
(PDF)

S1 Fig LFP time series and corresponding spectrograms. A typical 20 second epoch (following a high-dose ketamine bolus) of LFP from a single electrode of a multi-electrode array located in the frontal cortex (vlPFC) of NHP MJ is presented in panel A. Panel B shows the multitaper spectrogram for the same electrode, and panel C shows the average multitaper spectrogram for the vlPFC electrode array. Time 0 corresponds to 400 seconds after the ketamine bolus was administered. We fit our beta-HMM to the reduced-order representation of the corresponding spectrogram (derived using Eqs. (1) and (A1) in S1 Appendix), presented in panel C. The resulting optimal segmentation is represented by the colored vertical bars overlaid on the neural time-series in panel A. State 2 is shown in blue, state 3 in red, state 4 in yellow, and state 5 in green. (State 1, which corresponds to the time before the ketamine bolus, is not present in this epoch.).
(TIF)

S2 Fig NHP LFP spectrograms and corresponding state trajectories. Multitaper spectrograms of LFP and corresponding estimated latent state trajectories from 4 sessions in NHP MJ (A-D) and 5 sessions in NHP LM (E-I).
(TIF)

S3 Fig Human EEG spectrograms and corresponding state trajectories. Multitaper spectrograms of EEG and corresponding estimated latent state trajectories from all 9 human subjects (A-I). Note that in many patients, the EEG activity before ketamine was administered is less reliably distinguished from the EEG activity after. However, in most patients (A, B, C, D, F, I) there is a clear change in the state trajectories after ketamine is administered.
(TIF)

S4 Fig State- and frequency-specific beta pdfs for NHP MJ session 1. The state-specific and frequency-band specific beta pdfs corresponding to a 5-state beta-HMM were estimated from *L* = 1 session of LFP recording from NHP MJ. For each state (viewed row-wise) and a frequency band (viewed column-wise), the corresponding subplot presents (1) the empirical pdf plotted as a histogram based on the observations that correspond to the optimal segmentation of the data sequence, and (2) the continuous beta pdf with parameters estimated by the EM algorithm. Each color distinguishes a state as in Fig 3.
(TIF)

S5 Fig State- and frequency-specific beta pdfs for 4 NHP MJ sessions. The state-specific and frequency-band specific beta pdfs corresponding to a 5-state beta-HMM were estimated from *L* = 4 sessions of LFP recording from NHP MJ. For each state (viewed row-wise) and a frequency band (viewed column-wise), the corresponding subplot presents (1) the empirical pdf plotted as a histogram based on the observations that correspond to the optimal segmentation (Viterbi algorithm) across the L = 4 sessions, and (2) the continuous beta pdf with parameters estimated by the EM algorithm. Each color distinguishes a state as in Fig 4.
(TIF)

S6 Fig State- and frequency-specific beta pdfs for 5 NHP LM sessions. The state-specific and frequency-band specific beta pdfs corresponding to a 5-state beta-HMM were estimated from *L* = 5 sessions of LFP recording from NHP LM. For each state (viewed row-wise) and a frequency band (viewed column-wise), the corresponding subplot presents (1) the empirical pdf plotted as a histogram based on the observations that correspond to the optimal segmentation (Viterbi algorithm) across the L = 5 sessions, and (2) the continuous beta pdf with parameters estimated by the EM algorithm. Each color distinguishes a state as in Fig 4.
(TIF)

S7 Fig State- and frequency-specific beta pdfs for 9 human sessions. The state-specific and frequency-band specific beta pdfs corresponding to a 6-state beta-HMM were estimated from *L* = 9 sessions of EEG recording from 9 human OR patients. For each state (viewed row-wise) and a frequency band (viewed column-wise), the corresponding subplot presents (1) the empirical pdf plotted as a histogram based on the observations that correspond to the optimal segmentation (Viterbi algorithm) across the L = 9 sessions, and (2) the continuous beta pdf with parameters estimated by the EM algorithm. Each color distinguishes a state as in Fig 6.
(TIF)

S8 Fig Patient variability in state-wise mean scaled spectral power. In each state plot, one dot represents the mean scaled power in a specific frequency band for one patient, where the mean is calculated from the observations assigned to that state via the Viterbi algorithm. The mean scaled power across frequencies for each patient are connected with a line. The shaded region indicates the 95% confidence interval for the mean scaled power across 10000 samples of the mean of the corresponding beta distribution, where the mean is calculated from 200 independent samples of the beta distribution. Note that while some observed means exceed the 95% confidence interval of the expected mean of the corresponding estimated beta distribution, the overall trends are consistent across patients.
(TIF)

S9 Fig Exploration of how frequency resolution affects the outcome of the beta-HMM. Panels A and B present analysis from NHP MJ sessions 1–4, panels C and D present analysis from NHP LM sessions 1–5, and panels E and F present analysis from the 9 human EEG sessions. In panels A, C, and E, a portion of the spectrogram is displayed on the top, and two state trajectories are displayed below, estimated from models with H = 51 frequency bands (i.e. the highest possible frequency resolution equal to that of the estimated spectrogram) and H = 7* frequency bands (where 7* frequency bands indicate the canonical frequency bands described in Sec 3.2.2). Panels B, D, and F present the key duration statistics presented in Sec 4.3 and 4.4 across varying frequency-band resolution. With the exception of the models corresponding to 7*, the canonical frequency bands, all other models utilized evenly spaced frequency bands. For each model, 4000 Markov sequences of length *N* = 2000 were simulated using the estimated transition matrix. The mean duration and interval corresponding to the gamma and slow-delta activities were calculated for each realization of these sequences. Median and 95% confidence bounds across simulated sequences for models with equally spaced frequency bands are indicated in grey and, for the model utilizing canonical frequency bands, in blue. The mean duration and interval calculated from the estimated state trajectory (output of the Viterbi algorithm which uses the maximum likelihood beta-HMM parameters and the observations as input) are indicated by a cross (×) symbol. Note that the 95% confidence intervals of the durations across the varying frequency resolutions almost always overlap. This indicates that the specification of the frequency bands does not have a critical effect on the inferences related to the model dynamics. Thus, in the main manuscript, we chose to present the model estimated from canonical frequency bands that are commonly used to describe neural oscillations.
(TIF)

## References

[1] Domino EF, Chodoff P, Corssen G. Pharmacologic effects of CI-581, a new dissociative anesthetic, in man. Clinical Pharmacology & Therapeutics. 1965;6(3):279–291. doi: 10.1002/cpt196563279 14296024

[2] Domino EF. Taming the Ketamine Tiger. Anesthesiology: The Journal of the American Society of Anesthesiologists. 2010;113(3):678–684. doi: 10.1097/ALN.0b013e3181ed09a2 20693870

[3] Vuyk J, Sitsen E, Reekers M. Intravenous anesthetics. Miller’s anesthesia. 2015;8:858.

[4] WHO. Fact file on ketamine. World Health Organization; 2016.

[5] WHO. World Health Organization model list of essential medicines: 21st list 2019. World Health Organization; 2019.

[6] Bergman SA. Ketamine: review of its pharmacology and its use in pediatric anesthesia. Anesthesia progress. 1999;46(1):10. 10551055 PMC2148883

[7] Kurdi M, Theerth K, Deva R. Ketamine: Current applications in anesthesia, pain, and critical care. Anesthesia: Essays and Researches. 2014;8(3):283–290. doi: 10.4103/0259-1162.143110 25886322 PMC4258981

[8] Green SM, Roback MG, Kennedy RM, Krauss B. Clinical Practice Guideline for Emergency Department Ketamine Dissociative Sedation: 2011 Update. Annals of Emergency Medicine. 2011;57(5):449—461. doi: 10.1016/j.annemergmed.2010.11.030 21256625

[9] White PF, Schüttler J, Shafer A, Stanski DR, Horai Y, Trevor AJ, et al. Comparative Pharmacology of the Ketamine Isomers: Studies in Volunteers. BJA: British Journal of Anaesthesia. 1985;57(2):197–203. doi: 10.1093/bja/57.2.197 3970799

[10] Akeju O, Song AH, Hamilos AE, Pavone KJ, Flores FJ, Brown EN, et al. Electroencephalogram signatures of ketamine anesthesia-induced unconsciousness. Clinical neurophysiology. 2016;127(6):2414–2422. doi: 10.1016/j.clinph.2016.03.005 27178861 PMC4871620

[11] Li D, Mashour GA. Cortical dynamics during psychedelic and anesthetized states induced by ketamine. NeuroImage. 2019;196:32–40. doi: 10.1016/j.neuroimage.2019.03.076 30959192 PMC6559852

[12] Flores FJ, Ching S, Hartnack K, Fath AB, Purdon PL, Wilson MA, et al. A PK–PD model of ketamine-induced high-frequency oscillations. Journal of neural engineering. 2015;12(5):056006. doi: 10.1088/1741-2560/12/5/056006 26268223 PMC5764707

[13] Ahnaou A, Huysmans H, Biermans R, Manyakov N, Drinkenburg W. Ketamine: differential neurophysiological dynamics in functional networks in the rat brain. Translational psychiatry. 2017;7(9):e1237–e1237. doi: 10.1038/tp.2017.198 28926001 PMC5639243

[14] Castro-Zaballa S, Cavelli ML, Gonzalez J, Nardi AE, Machado S, Scorza C, et al. EEG 40 Hz coherence decreases in REM sleep and ketamine model of psychosis. Frontiers in psychiatry. 2019;9:766. doi: 10.3389/fpsyt.2018.00766 30705645 PMC6345101

[15] Ballesteros JJ, Huang P, Patel SR, Eskandar EN, Ishizawa Y. Dynamics of Ketamine-induced Loss and Return of Consciousness across Primate Neocortex. Anesthesiology. 2020;132(4):750–762. doi: 10.1097/ALN.0000000000003159 32053559

[16] Schroeder KE, Irwin ZT, Gaidica M, Bentley JN, Patil PG, Mashour GA, et al. Disruption of corticocortical information transfer during ketamine anesthesia in the primate brain. NeuroImage. 2016;. doi: 10.1016/j.neuroimage.2016.04.039 27095309 PMC4912854

[17] Slovik M, Rosin B, Moshel S, Mitelman R, Schechtman E, Eitan R, et al. Ketamine induced converged synchronous gamma oscillations in the cortico-basal ganglia network of nonhuman primates. Journal of neurophysiology. 2017;118(2):917–931. doi: 10.1152/jn.00765.2016 28468999 PMC5539445

[18] Nicol AU, Morton AJ. Characteristic patterns of EEG oscillations in sheep (Ovis aries) induced by ketamine may explain the psychotropic effects seen in humans. Scientific Reports. 2020;10(1):1–10. doi: 10.1038/s41598-020-66023-8 32528071 PMC7289807

[19] Zanos P, Moaddel R, Morris PJ, Riggs LM, Highland JN, Georgiou P, et al. Ketamine and Ketamine Metabolite Pharmacology: Insights into Therapeutic Mechanisms. Pharmacological Reviews. 2018;70(3):621–660. doi: 10.1124/pr.117.015198 29945898 PMC6020109

[20] Seamans J. Losing inhibition with ketamine. Nature chemical biology. 2008;4(2):91–93. doi: 10.1038/nchembio0208-91 18202677

[21] Homayoun H, Moghaddam B. NMDA Receptor Hypofunction Produces Opposite Effects on Prefrontal Cortex Interneurons and Pyramidal Neurons. Journal of Neuroscience. 2007;27(43):11496–11500. doi: 10.1523/JNEUROSCI.2213-07.2007 17959792 PMC2954603

[22] Kowalski MM, Donoghue JA, McCarthy MM, Kopell NJ, Miller EK, Brown EN, et al. Ketamine anesthesia produces alternating peaks in delta and gamma power in prefrontal and parietal cortex of macaque monkeys. Program No 75113 2017 Neuroscience Meeting Planner San Diego, IL: Society for Neuroscience. 2017;.

[23] McCarthy MM, Brown EN, Kopell N. Potential network mechanisms mediating electroencephalographic beta rhythm changes during propofol-induced paradoxical excitation. Journal of Neuroscience. 2008;28(50):13488–13504. doi: 10.1523/JNEUROSCI.3536-08.2008 19074022 PMC2717965

[24] Ching S, Cimenser A, Purdon PL, Brown EN, Kopell NJ. Thalamocortical model for a propofol-induced *α*-rhythm associated with loss of consciousness. Proceedings of the National Academy of Sciences. 2010;107(52):22665–22670. doi: 10.1073/pnas.1017069108 21149695 PMC3012501

[25] Vijayan S, Ching S, Purdon PL, Brown EN, Kopell NJ. Thalamocortical Mechanisms for the Anteriorization of Alpha Rhythms during Propofol-Induced Unconsciousness. Journal of Neuroscience. 2013;33(27):11070–11075. doi: 10.1523/JNEUROSCI.5670-12.2013 23825412 PMC3718379

[26] Soplata AE, McCarthy MM, Sherfey J, Lee S, Purdon PL, Brown EN, et al. Thalamocortical control of propofol phase-amplitude coupling. PLoS computational biology. 2017;13(12):e1005879. doi: 10.1371/journal.pcbi.1005879 29227992 PMC5739502

[27] Kass RE, Eden UT, Brown EN. Analysis of neural data. vol. 491. Springer; 2014.

[28] Brown EN, Frank LM, Tang D, Quirk MC, Wilson MA. A statistical paradigm for neural spike train decoding applied to position prediction from ensemble firing patterns of rat hippocampal place cells. Journal of Neuroscience. 1998;18(18):7411–7425. doi: 10.1523/JNEUROSCI.18-18-07411.1998 9736661 PMC6793233

[29] Churchland MM, Cunningham JP, Kaufman MT, Foster JD, Nuyujukian P, Ryu SI, et al. Neural population dynamics during reaching. Nature. 2012;487(7405):51–56. doi: 10.1038/nature11129 22722855 PMC3393826

[30] Hochberg LR, Serruya MD, Friehs GM, Mukand JA, Saleh M, Caplan AH, et al. Neuronal ensemble control of prosthetic devices by a human with tetraplegia. Nature. 2006;442(7099):164–171. doi: 10.1038/nature04970 16838014

[31] Chestek CA, Gilja V, Nuyujukian P, Foster JD, Fan JM, Kaufman MT, et al. Long-term stability of neural prosthetic control signals from silicon cortical arrays in rhesus macaque motor cortex. Journal of Neural Engineering. 2011;8(4). doi: 10.1088/1741-2560/8/4/045005 21775782 PMC3644617

[32] Chemali J, Ching S, Purdon PL, Solt K, Brown EN. Burst suppression probability algorithms: state-space methods for tracking EEG burst suppression. Journal of Neural Engineering. 2013;10(5):1–20. doi: 10.1088/1741-2560/10/5/056017 24018288 PMC3793904

[33] Bishop CM. Pattern recognition and machine learning. springer; 2006.

[34] Särkkä S. Bayesian filtering and smoothing. vol. 3. Cambridge University Press; 2013.

[35] Rabiner LR. A tutorial on hidden Markov models and selected applications in speech recognition. Proceedings of the IEEE. 1989;77(2):257–286. doi: 10.1109/5.18626

[36] Liu Z, Huang J, Wang Y. Classification TV programs based on audio information using hidden Markov model. In: 1998 IEEE Second Workshop on Multimedia Signal Processing (Cat. No.98EX175); 1998. p. 27–32.

[37] Mysore GJ, Smaragdis P, Raj B. Non-negative Hidden Markov Modeling of Audio with Application to Source Separation. In: Vigneron V, Zarzoso V, Moreau E, Gribonval R, Vincent E, editors. Latent Variable Analysis and Signal Separation. Berlin, Heidelberg: Springer Berlin Heidelberg; 2010. p. 140–148.

[38] Mustafa MK, Allen T, Appiah K. A comparative review of dynamic neural networks and hidden Markov model methods for mobile on-device speech recognition. Neural Computing and Applications. 2019;31(2):891–899. doi: 10.1007/s00521-017-3028-2

[39] Lee M, Youn I, Ryu J, Kim DH. Classification of Both Seizure and Non-Seizure Based on EEG Signals Using Hidden Markov Model. Proceedings—2018 IEEE International Conference on Big Data and Smart Computing, BigComp 2018. 2018; p. 469–474.

[40] Ghimatgar H, Kazemi K, Helfroush MS, Aarabi A. An automatic single-channel EEG-based sleep stage scoring method based on hidden Markov Model. Journal of Neuroscience Methods. 2019;324(June):108320. doi: 10.1016/j.jneumeth.2019.108320 31228517

[41] Doroshenkov LG, Konyshev VA, Selishchev SV. Classification of Human Sleep Stages Based on EEG Processing Using Hidden Markov Models. Biomedical Engineering. 2007;41(1):25–28. doi: 10.1007/s10527-007-0006-5 17419342

[42] Flexer A, Dorffner G, Sykacek P, Rezek I. An automatic, continuous and probabilistic sleep stager based on a Hidden Markov Model. Applied Artificial Intelligence. 2002;16(3):199–207. doi: 10.1080/088395102753559271 15811785

[43] Song AH, Chlon L, Soulat H, Tauber J, Subramanian S, Ba D, et al. Multitaper Infinite Hidden Markov Model for EEG. In: 2019 41st Annual International Conference of the IEEE Engineering in Medicine and Biology Society (EMBC). IEEE; 2019. p. 5803–5807. 10.1109/EMBC.2019.8856817 PMC7029542 31947171

[44] Bryan JD, Levinson SE. Autoregressive Hidden Markov Model and the Speech Signal. Procedia Computer Science. 2015;61:328–333. doi: 10.1016/j.procs.2015.09.151

[45] Moses DA, Leonard MK, Makin JG, Chang EF. Real-time decoding of question-and-answer speech dialogue using human cortical activity. Nature Communications. 2019;10(1). doi: 10.1038/s41467-019-10994-4 31363096 PMC6667454

[46] Kao JC, Nuyujukian P, Ryu SI, Shenoy KV. A High-Performance Neural Prosthesis Incorporating Discrete State Selection With Hidden Markov Models. IEEE Transactions on Biomedical Engineering. 2017;64(4):935–945. doi: 10.1109/TBME.2016.2582691 27337709

[47] Lederman D, Tabrikian J. Classification of multichannel EEG patterns using parallel hidden markov models. Medical and Biological Engineering and Computing. 2012;50(4):319–328. doi: 10.1007/s11517-012-0871-2 22407476

[48] Chen Z, Vijayan S, Barbieri R, Wilson MA, Brown EN. Discrete- and continuous-time probabilistic models and algorithms for inferring neuronal UP and DOWN states. Neural computation. 2009;21(7):1797–1862. doi: 10.1162/neco.2009.06-08-799 19323637 PMC2799196

[49] Mcfarland JM, Hahn TTG, Mehta MR. Explicit-Duration Hidden Markov Model Inference of UP-DOWN States from Continuous Signals. PLOS ONE. 2011;6(6). doi: 10.1371/journal.pone.0021606 21738730 PMC3125293

[50] Tokdar S, Xi P, Kelly RC, Kass RE. Detection of bursts in extracellular spike trains using hidden semi-Markov point process models. Journal of Computational Neuroscience. 2010;29(1-2):203–212. doi: 10.1007/s10827-009-0182-2 19697116

[51] Rice IC, Chakravarty S, Kahali P, Donoghue J, Mahnke M, Miller EK, et al. Detecting bursts in electroencephalography and local field potential spectrograms using a hidden Markov model. Program No 52312 2018 Neuroscience Meeting Planner San Diego, IL: Society for Neuroscience. 2018;.

[52] Vidaurre D, Quinn AJ, Baker AP, Dupret D, Tejero-Cantero A, Woolrich MW, et al. Spectrally resolved fast transient brain states in electrophysiological data. Neuroimage. 2016;126:81–95. doi: 10.1016/j.neuroimage.2015.11.047 26631815 PMC4739513

[53] Purdon PL, Sampson A, Pavone KJ, Brown EN. Clinical Electroencephalography for Anesthesiologists: Part I: Background and Basic Signatures. Anesthesiology. 2015;123(4):937–960. doi: 10.1097/ALN.0000000000000841 26275092 PMC4573341

[54] Thomson DJ. Spectrum estimation and harmonic analysis. Proceedings of the IEEE. 1982;70(9):1055–1096. doi: 10.1109/PROC.1982.12433

[55] Mitra P. Observed brain dynamics. Oxford University Press; 2007.

[56] Bokil H, Andrews P, Kulkarni JE, Mehta S, Mitra PP. Chronux: A platform for analyzing neural signals. Journal of Neuroscience Methods. 2010;192(1):146—151. doi: 10.1016/j.jneumeth.2010.06.020 20637804 PMC2934871

[57] Rosenthal JS. First Look At Rigorous Probability Theory, A. World Scientific Publishing Company; 2006.

[58] Cook JD, Nadarajah S. Stochastic Inequality Probabilities for Adaptively Randomized Clinical Trials. Biometrical Journal. 2006;48(3):356–365. doi: 10.1002/bimj.200510220 16845901

[59] Unit for Laboratory Animal Medicine U. of Michigan Guidelines on Anesthesia and Analgesia in Non-Human Primates; 2017.

[60] Bertrand HG, Ellen YC, O’Keefe S, Flecknell PA. Comparison of the effects of ketamine and fentanyl-midazolam-medetomidine for sedation of rhesus macaques (Macaca mulatta). BMC Veterinary Research. 2016;12(1):1–9. doi: 10.1186/s12917-016-0721-9 27277424 PMC4898395

[61] Hawk CT, Leary SL, Morris TH. Formulary for laboratory animals. 3rd ed. Blackwell Publishing Professional; 2005.

[62] Kim SE, Behr MK, Ba D, Brown EN. State-space multitaper time-frequency analysis. Proceedings of the National Academy of Sciences. 2018;115(1):E5–E14. doi: 10.1073/pnas.1702877115 29255032 PMC5776784

[63] Song AH, Chakravarty S, Brown EN. A Smoother State Space Multitaper Spectrogram. In: 2018 40th Annual International Conference of the IEEE Engineering in Medicine and Biology Society (EMBC); 2018. p. 33–36. 10.1109/EMBC.2018.8512190 30440334

[64] Soulat H, Stephen EP, Beck AM, Purdon PL. State Space Methods for Phase Amplitude Coupling Analysis. bioRxiv. 2019;. 10.1038/s41598-022-18475-3 PMC9509338 36153353

[65] Yousefi A, Fard RS, Eden UT, Brown EN. State-Space Global Coherence to Estimate the Spatio-Temporal Dynamics of the Coordinated Brain Activity. In: 2019 41st Annual International Conference of the IEEE Engineering in Medicine and Biology Society (EMBC); 2019. p. 5794–5798. 10.1109/EMBC.2019.8856634 31947169

[66] Prerau MJ, Brown RE, Bianchi MT, Ellenbogen JM, Purdon PL. Sleep neurophysiological dynamics through the lens of multitaper spectral analysis. Physiology. 2017;32(1):60–92. doi: 10.1152/physiol.00062.2015 27927806 PMC5343535

[67] Brown EN, Lydic R, Schiff ND. General anesthesia, sleep, and coma. New England Journal of Medicine. 2010;363(27):2638–2650. doi: 10.1056/NEJMra0808281 21190458 PMC3162622

[68] Prerau MJ, Purdon PL. A probabilistic framework for time-frequency detection of burst suppression. In: 2013 6th International IEEE/EMBS Conference on Neural Engineering (NER). IEEE; 2013. p. 609–612.

[69] Cannon J, McCarthy MM, Lee S, Lee J, Börgers C, Whittington MA, et al. Neurosystems: brain rhythms and cognitive processing. European Journal of Neuroscience. 2014;39(5):705–719. doi: 10.1111/ejn.12453 24329933 PMC4916881

[70] McCarthy MM, Ching S, Whittington MA, Kopell N. Dynamical changes in neurological diseases and anesthesia. Current Opinion in Neurobiology. 2012;22(4):693—703. doi: 10.1016/j.conb.2012.02.009 22446010 PMC3965179

[71] Zarate CA Jr, Singh JB, Carlson PJ, Brutsche NE, Ameli R, Luckenbaugh DA, et al. A Randomized Trial of an N-methyl-D-aspartate Antagonist in Treatment-Resistant Major Depression. Archives of General Psychiatry. 2006;63(8):856–864. doi: 10.1001/archpsyc.63.8.856 16894061

[72] Salvadore G, Singh JB. Ketamine as a Fast Acting Antidepressant: Current Knowledge and Open Questions. CNS Neuroscience & Therapeutics. 2013;19(6):428–436. doi: 10.1111/cns.12103 23578128 PMC6493512

[73] Insel TR. Rethinking schizophrenia. Nature. 2010;468(7321):187–193. doi: 10.1038/nature09552 21068826

[74] Frohlich J, Horn JDV. Reviewing the ketamine model for schizophrenia. Journal of Psychopharmacology. 2014;28(4):287–302. doi: 10.1177/0269881113512909 24257811 PMC4133098

[75] Vesuna S, Kauvar IV, Richman E, Gore F, Oskotsky T, Sava-Segal C, et al. Deep posteromedial cortical rhythm in dissociation. Nature. 2020;586(7827):87–94. doi: 10.1038/s41586-020-2731-9 32939091 PMC7553818

[76] Purdon PL, Pierce ET, Mukamel EA, Prerau MJ, Walsh JL, Wong KFK, et al. Electroencephalogram signatures of loss and recovery of consciousness from propofol. Proceedings of the National Academy of Sciences. 2013;110(12):E1142–E1151. doi: 10.1073/pnas.1221180110 23487781 PMC3607036

[77] Ishizawa Y, Ahmed OJ, Patel SR, Gale JT, Sierra-Mercado D, Brown EN, et al. Dynamics of propofol-induced loss of consciousness across primate neocortex. Journal of Neuroscience. 2016;36(29):7718–7726. doi: 10.1523/JNEUROSCI.4577-15.2016 27445148 PMC4951576

[78] Moyal R, Edelman S. Dynamic computation in visual thalamocortical networks. Entropy. 2019;21(5):500. doi: 10.3390/e21050500 33267214 PMC7514988

[79] Varley TF, Sporns O, Puce A, Beggs J. Differential effects of propofol and ketamine on critical brain dynamics. PLOS Computational Biology. 2020;16(12):e1008418. doi: 10.1371/journal.pcbi.1008418 33347455 PMC7785236

[80] Barttfeld P, Uhrig L, Sitt JD, Sigman M, Jarraya B, Dehaene S, et al. Signature of consciousness in the dynamics of resting-state brain activity. Proceedings of the National Academy of Sciences. 2015;112(3):887–892. doi: 10.1073/pnas.1418031112 25561541 PMC4311826

[81] Chennu S, O’Connor S, Adapa R, Menon DK, Bekinschtein TA. Brain connectivity dissociates responsiveness from drug exposure during propofol-induced transitions of consciousness. PLoS computational biology. 2016;12(1):e1004669. doi: 10.1371/journal.pcbi.1004669 26764466 PMC4713143

