# Supplementary material for: A hidden Markov model reliably characterizes ketamine-induced spectral dynamics in macaque local field potentials and human electroencephalograms
Source: PLoS Comput Biol. 2021 Aug 18;17(8):e1009280. doi: 10.1371/journal.pcbi.1009280 (PMC8405019; doi:10.1371/journal.pcbi.1009280)
Supplement: S1 Appendix — We also provide the algorithm for simulating a spectrogram with known Markov model parameters (Sec A5, Table A2), and a tutorial on interpreting beta distributions (Sec A6). In Table A1, we provide a glossary of mathematical symbols. (PDF) [file pcbi.1009280.s001.pdf]

A hidden Markov model reliably characterizes ketamine-induced spectral dynamics in macaque local field potentials and human electroencephalograms

Indie C. Garwood[#,⋆, 1,2,3], Sourish Chakravarty[#, 2, 4], Jacob Donoghue[1,2,3], Meredith Mahnke[2], Pegah Kahali[2,4], Shubham Chamadia[4], Oluwaseun Akeju[4], Earl K. Miller[2,3], and Emery N. Brown[†, 1,2,3,4]

[1]Harvard-MIT Division of Health Sciences and Technology, Massachusetts Institute of Technology, Cambridge, Massachusetts, USA; [2]The Picower Institute for Learning and Memory, Massachusetts Institute of Technology, Cambridge, Massachusetts, USA; [3]Department of Brain and Cognitive Sciences, Massachusetts Institute of Technology, Cambridge, Massachusetts, USA; [4]Department of Anesthesia, Critical Care, and Pain Medicine, Massachusetts General Hospital, Boston, Massachusetts, USA; [#]Co-first authors; [†]Senior author; [⋆]garwood@mit.edu

S1 Appendix

In this appendix, we provide additional detail in the calculation of band-wise spectral power (Sec A1), scaling of the beta-HMM observations (Sec A2), estimation of model parameters (Sec A3), and EM algorithm (Sec A4). We also provide the algorithm for simulating a spectrogram with known Markov model parameters (Sec A5), and a tutorial on interpreting beta distributions (Sec A6). In Table A1, we provide a glossary of mathematical symbols.

A1 Band-wise power

We reduce the spectrogram observations, $S_n(\{\omega_i\})$, to a low-dimensional vector $\bar{S}_n(\{\omega_h\})$. This vector characterizes the instantaneous average power in H $(= 7)$ distinct frequency bands between 0-50 Hz, such that

$$\bar{S}_n(\omega_h) = \frac{\omega_r}{\delta_h} \sum_{i=\delta_h\omega_r^{-1}(h-1)+1}^{\delta_h\omega_r^{-1}h} S_n(\omega_i) \ , \quad \text{where } h \in \{1, 2, \cdots, H\}, \tag{A1}$$

where ω_r denotes the frequency resolution of the multitaper spectral estimates and δ_h denotes the band-width of the h'th frequency band.

A2 Observation scaling

The goal of our band-wise spectral observation scaling approach is to scale the observations between [0,1] while maintaining a large dynamic range in the observations. A strictly linear scaling approach would be highly sensitive to outliers and result in low variance observations. Thus, we sought to scale the majority of the band-wise power observations approximately linearly between [0,1], but to non-linearly scale the observations near the extrema of the data such that very small values are close to zero and very large values are close to one. Our scaling approach is based on the logistic function and an assumption that the first and third quartiles are approximately equidistant from the median. Note that the logistic function, $y_{nh}(x_{nh}) = 1/(1 + \exp(-\lambda_h^* x_{nh})$, where $x_{nh} = (\bar{S}_n(\omega_h)) - Q_2(\bar{\boldsymbol{S}}(\omega_h)))/(Q_3(\bar{\boldsymbol{S}}(\omega_h)) - Q_1(\bar{\boldsymbol{S}}(\omega_h)))$, is approximately linear between $y_{nh}(x_{nh}) = [0.25, 0.75]$. Thus, it is an intuitive choice for the first, second, and third quartile to be scaled to 0.25, 0.5, and 0.75, respectively. Using the assumption that the first and third quartiles are equidistant from the second quartile (the median), we can solve for the parameter λ_h^*, where $x_{nh} =$

Table A1: Glossary of mathematical symbols.

F_s	Sampling frequency (in Hz)
Δ_{MT}	Length of the time-window (in seconds) over which the signal is assumed to be second order stationary.
$S_n(\omega_i)$	Power spectral density (in dB) corresponding to frequency ω_i (in Hz) at the n-th time-window.
ω_r	Frequency resolution (in Hz) at which the multitaper spectral estimates are calculated.
$\bar{S}_n(\omega_h)$	Average power spectral density for the h-th frequency band. Note: we use the symbol ω to indicate both frequency and frequency-band. The context makes the distinction clear.
H	Number (num.) of frequency bands.
N	Num. of time-points where spectral estimates are available from a neural (LFP or EEG) recording session.
$\boldsymbol{\omega}$	$= \{\omega_h\}_{h=1}^{H}$, the set of all frequency bands,
$\bar{\boldsymbol{S}}(\omega_h)$	$= \{\bar{S}_n(\omega_h)\}_{n=1}^{N}$, vector of N average spectral densities for the frequency band ω_h.
$Q_j(\bar{\boldsymbol{S}}(\omega_h))$	The j-th quartile of the sequence $\bar{\boldsymbol{S}}(\omega_h)$
$\boldsymbol{y}_n$	Vector (of size $H \times 1$) scaled power values corresponding to the n-th time-point.
$\boldsymbol{y}_h$	Vector (of size $N \times 1$) scaled power values corresponding to the h-th frequency band.
y_{nh}	The h-th element of $\boldsymbol{y}_n$ taking real values in the range [0,1].
λ_h	A user-defined parameter used in scaling the spectral power values in the h-th frequency band to a value in the range $[0, 1]$.
K	Num. of latent states in a HMM.
z_n	Discrete-valued random variable denoting the latent state at the n-th timepoint. $z_n \in [1, K]$.
$p(x)$	The value that the probability density function (pdf) takes at a sample point x of a continuous-valued random variable. The corresponding probability space is implied [1].
$\mathcal{B}(a, b)$	The beta function taking arguments a and b.
a_{hk}, b_{hk}	The parameters of the beta pdf corresponding to the k-th latent state and h-th frequency band.
ϕ_k	The set of beta pdf parameters corresponding to the k-th latent state and all the H frequency bands $= \{a_{hk}, b_{hk}\}_{h=1}^{H}$.
$\boldsymbol{\phi}$	The set of all beta pdf parameters corresponding to all the K latent states and all the H frequency bands $= \{\phi_k\}_{k=1}^{K}$.
$Pr(\cdot)$	The probability measure that maps an element in the event space (corresponding to a random variable) to a value in the interval $[0, 1]$ [1].
A_{jk}	Probability to transition to state $z_n = k$ from a previous state $z_{n-1} = j$ for any $n \in [2, N]$.
$\boldsymbol{A}$	The $K \times K$ state transition matrix whose element in the j-th row and k-th column is A_{jk}.
π_k	Probability of the occurrence $z_1 = k$ where z_1 indicates the initial state of a Markov path.
$\boldsymbol{\pi}$	$= \{\pi_k\}_{k=1}^{K}$, the vector of initial state probabilities
L	Num. of statistically independent recording sessions that a model will be fitted to.
$\boldsymbol{y}_{n,l}$	The observation vector at the n-th time-point of the l-th recording session.
N_l	Num. of time-points where spectral estimates are available from the l-th neural recording session.
$\boldsymbol{Y}_l$	$= \{\boldsymbol{y}_{n,l}\}_{n=1}^{N_l}$, the set of all scaled powers from the l-th neural recording session.
$\boldsymbol{\pi}_l$	The initial state probability vector for the l-th recording session.
Θ	Set of all model parameters, $\{\{\boldsymbol{\pi}_l\}_{l=1}^{L}, \boldsymbol{A}, \boldsymbol{\phi}\}$, of a HMM describing the observations in L independent recording sessions.
$z_{n,l}$	The latent state at the n-th time-point of the l-th session.
$\boldsymbol{Z}_l$	$= \{z_{n,l}\}_{n=1}^{N_l}$, the set of N_l latent states denoting the latent state trajectory for the l-th session .
$Q(\Theta, \widehat{\Theta})$	The expectation of the complete data log-likelihood function (parameterized by Θ) where the expectation operation is over latent state trajectories, $\boldsymbol{Z}_1, \cdots, \boldsymbol{Z}_L$, that are estimated using an HMM with parameters $\widehat{\Theta}$.
$\gamma_{n,l}(k)$	$= Pr(z_{n,l} = k \mid \boldsymbol{Y}_l, \boldsymbol{\pi}, \boldsymbol{A}, \boldsymbol{\phi})$, the posterior probability of the occurrence of $z_{n,l} = k$ given the observations from the l-th session ($\boldsymbol{Y}_l$) and model parameters ($\boldsymbol{\pi}$, $\boldsymbol{A}$ and $\boldsymbol{\phi}$)).
$\xi_{n,l}(j, k)$	$= Pr(z_{n-1,l} = j, z_{n,l} = k \mid \boldsymbol{Y}_l, \boldsymbol{\pi}, \boldsymbol{A}, \boldsymbol{\phi})$, the posterior probability of the joint occurrence of $z_{n-1,l} = j$ and $z_{n,l} = k$ given the observations from the l-th session ($\boldsymbol{Y}_l$) and model parameters ($\boldsymbol{\pi}$, $\boldsymbol{A}$, $\boldsymbol{\phi}$).
$(Y_h \mid Z = k)$	Scalar-valued random variable indicating the scaled power Y_h in the h-th frequency band conditioned on the discrete-valued random variable Z, the latent state, at k.
Δ_{hjk}	A random variable denoting the difference between two scalar-valued continuous random variables as such, $X_{hj} - X_{hk}$, where $X_{hj} \sim Beta(a_{hj}, b_{hj})$ and $X_{hk} \sim Beta(a_{hk}, b_{hk})$.

$(Q_1(\bar{\boldsymbol{S}}(\omega_h)) - Q_2(\bar{\boldsymbol{S}}(\omega_h)))/(Q_3(\bar{\boldsymbol{S}}(\omega_h)) - Q_1(\bar{\boldsymbol{S}}(\omega_h))) = -1/2$ and $y_{nh}(x_{nh}) = 0.25$. This results in $\lambda_h^* = 2\log(3)$. Note that if $\lambda_h^* = 2\log(3)$ and $x_{nh} = (Q_3(\bar{\boldsymbol{S}}(\omega_h)) - Q_2(\bar{\boldsymbol{S}}(\omega_h)))/(Q_3(\bar{\boldsymbol{S}}(\omega_h)) - Q_1(\bar{\boldsymbol{S}}(\omega_h))) = 1/2$, then $y_{nh}(x_{nh}) = 0.75$. Finally, when $x_{nh} = (Q_2(\bar{\boldsymbol{S}}(\omega_h)) - Q_2(\bar{\boldsymbol{S}}(\omega_h)))/(Q_3(\bar{\boldsymbol{S}}(\omega_h)) - Q_1(\bar{\boldsymbol{S}}(\omega_h))) = 0$, $y_{nh}(x_{nh}) = 0.5$. Thus, the scaling of spectral power such that

$$y_{nh} = \frac{1}{1 + e^{-\lambda_h(\bar{S}_n(\omega_h) - Q_2(\bar{\boldsymbol{S}}(\omega_h)))}} \text{ , for } h \in \{1, \cdots, H\} \text{ ,} \tag{A2}$$

where $\lambda_h = 2\log(3)/(Q_3(\bar{\boldsymbol{S}}(\omega_h)) - Q_1(\bar{\boldsymbol{S}}(\omega_h)))$, results in approximately linear scaling of the band-wise power, $\bar{\boldsymbol{S}}(\omega_h)$, between the first and third quartiles to $[0.25, 0.75]$. The median of $\bar{\boldsymbol{S}}(\omega_h)$ is scaled to 0.5.

A3 Estimation of states and model parameters

We determined the model parameters of the beta-HMM from one or more single channel LFP or EEG LFP recordings using the maximum likelihood approach via the well-established Expectation-Maximization (EM) algorithm [2–5]. For a given set of L separate recording sessions, $\{\boldsymbol{Y}_1, \cdots, \boldsymbol{Y}_L\}$, where the l-th session is denoted by $\boldsymbol{Y}_l = \{\boldsymbol{y}_{n,l}\}_{n=1}^{N_l}$, we assume that data can be described by an HMM associated with the parameter set, $\Theta = \{\{\boldsymbol{\pi}_l\}_{l=1}^{L}, \boldsymbol{A}, \boldsymbol{\phi}\}$. Thus, we assume that the transition matrix, $\boldsymbol{A}$, and the state-specific beta distribution parameters, $\boldsymbol{\phi} = \{\boldsymbol{\phi}_k\}_{k=1}^{K}$, remain constant across all L sessions, but the initial state probability $\boldsymbol{\pi}_l$ can vary across the sessions. When $L = 1$ the problem reduces to learning a beta-HMM using data from a single recording session as demonstrated in Sec 4.2 with an LFP recording session from an NHP. In this work (as shown in Sec 4.3) we also learn subject specific beta-HMMs for 2 NHPs using $L = 4$ separate recording LFP sessions from one NHP, and $L = 5$ from the other. As shown in Sec 4.4 we also learn a beta-HMM for a typical patient using $L = 9$ independent EEG recording sessions, each from a separate human subject. For $L \geq 1$ mutually independent recording sessions, the complete data likelihood for a given Θ can be written as,

$$\begin{aligned}
p(\{\boldsymbol{Y}_1, \boldsymbol{Z}_1, \cdots, \boldsymbol{Y}_L, \boldsymbol{Z}_L\}|\Theta) &= \prod_{l=1}^{L} p(\boldsymbol{Y}_l, \boldsymbol{Z}_l|\boldsymbol{\pi}_l, \boldsymbol{A}, \boldsymbol{\phi}) \\
&= \prod_{l=1}^{L} \left(Pr(z_{1,l}|\boldsymbol{\pi}_l) \prod_{n=2}^{N_l} Pr(z_{n,l}|z_{n-1,l}, \boldsymbol{A}) \prod_{n=1}^{N_l} p(\boldsymbol{y}_{n,l}|z_{n,l} = k; \boldsymbol{\phi}_k) \right) \\
&= \prod_{l=1}^{L} \left(\pi_{z_{1,l}} \prod_{n=2}^{N_l} A_{z_{n-1,l} z_{n,l}} \prod_{n=1}^{N_l} \prod_{h=1}^{H} p(y_{nh,l}|z_{n,l} = k; a_{hk}, b_{hk}) \right)
\end{aligned} \tag{A3}$$

where subscript $(\cdot)_{,l}$ denotes the random processes corresponding to the data sequence $\boldsymbol{Y}_l$. In the above expression, $\boldsymbol{Z}_l \equiv \{z_{1,l}, \cdots, z_{N_l,l}\}$ denotes the sequence of latent states corresponding to $\boldsymbol{Y}_l$. In the ensuing discussion, we shall refer to the expression, $\log(p(\{\boldsymbol{Y}_1, \boldsymbol{Z}_1, \cdots, \boldsymbol{Y}_L, \boldsymbol{Z}_L\}|\Theta))$, as the complete data log-likelihood. Note that by marginalizing over all possible $\{\boldsymbol{Z}_1, \cdots, \boldsymbol{Z}_L\}$, we are able to obtain the likelihood of $\{\boldsymbol{Y}_1, \cdots, \boldsymbol{Y}_L\}$ given the model, $p(\{\boldsymbol{Y}_1, \cdots, \boldsymbol{Y}_L\}|\Theta)$.

The structure of the likelihood expression (Eq. (A3)) allows for the maximum likelihood beta-HMM to be estimated in terms of the parameter set $\Theta = \{\{\boldsymbol{\pi}_l\}_{l=1}^{L}, \boldsymbol{A}, \boldsymbol{\phi}\}$ using the EM algorithm [2–5]. The EM algorithm is commonly used for HMMs (the case of $L = 1$ being the most familiar case [3]) with observation distributions from the exponential family (e.g. the Gaussian distribution) [2, 3, 6, 7]. Briefly, the standard EM algorithm is an iterative optimization scheme where in each EM iteration, we perform an E-step followed by an M-step. In the E-step, following [2, Eq. 13.17], we run L independent *Forward-Backward*

algorithms ([2,3]) to estimate the sequences $\{\boldsymbol{\gamma}_{n,l}\}_{n=1}^{N_l} \equiv \{Pr(z_{n,l} = k|\boldsymbol{Y}_l, \widehat{\boldsymbol{\pi}}_l, \widehat{\boldsymbol{A}}, \widehat{\boldsymbol{\phi}})\}_{n=1}^{N_l}$ (i.e. the posterior probability of each state at each time-point) and $\{\boldsymbol{\xi}_{n,l}\}_{n=1}^{N_l} \equiv \{Pr(z_{n-1,l} = j, z_{n,l} = k|\boldsymbol{Y}_l, \widehat{\boldsymbol{\pi}}_l, \widehat{\boldsymbol{A}}, \widehat{\boldsymbol{\phi}})\}_{n=1}^{N_l}$ (i.e. the posterior probability of each state transition) given the observations ($\{\boldsymbol{Y}_1, \cdots, \boldsymbol{Y}_L\}$) and the last best estimate of the model parameters ($\widehat{\Theta} = \{\{\widehat{\boldsymbol{\pi}}_l\}_{l=1}^{L}, \widehat{\boldsymbol{A}}, \widehat{\boldsymbol{\phi}})\}$) from the previous iteration. Note that the likelihood value, $p(\{\boldsymbol{Y}_1, \cdots, \boldsymbol{Y}_L|\widehat{\Theta}\})$, can also be readily calculated following the forward-step of the Forward-Backward algorithm [2, 3].

The M-step of each iteration of the EM algorithm is posed as a maximization (over the space of allowable parameters Θ) of the *expectation* of the complete data log-likelihood function, denoted by $Q(\Theta, \widehat{\Theta})$ and defined as,

$$\begin{aligned}
Q(\Theta, \widehat{\Theta}) &= \mathbb{E}_{\{\boldsymbol{Z}_1,\cdots,\boldsymbol{Z}_L\}|\{\boldsymbol{Y}_1,\cdots,\boldsymbol{Y}_L\},\widehat{\Theta}} \left[\log\left(\prod_{l=1}^{L} p(\boldsymbol{Y}_l, \boldsymbol{Z}_l|\Theta)\right)\right] \\
&= \sum_{\{\boldsymbol{Z}_1,\cdots,\boldsymbol{Z}_L\}} Pr(\{\boldsymbol{Z}_1, \cdots, \boldsymbol{Z}_L\}|\{\boldsymbol{Y}_1, \cdots, \boldsymbol{Y}_L\}, \widehat{\Theta}) \sum_{l=1}^{L} \log p(\boldsymbol{Y}_l, \boldsymbol{Z}_l|\Theta) \\
&= \sum_{l=1}^{L} \sum_{\boldsymbol{Z}_l} Pr(\boldsymbol{Z}_l|\boldsymbol{Y}_l, \widehat{\Theta}) \log p(\boldsymbol{Y}_l, \boldsymbol{Z}_l|\Theta) \\
&= \sum_{l=1}^{L} \sum_{k=1}^{K} \gamma_{1,l}(k) \log \pi_{k,l} + \sum_{l=1}^{L} \sum_{n=2}^{N_l} \sum_{j=1}^{K} \sum_{k=1}^{K} \xi_{n,l}(j,k) \log A_{jk} \\
&+ \sum_{l=1}^{L} \sum_{n=1}^{N_l} \sum_{k=1}^{K} \gamma_{n,l}(k) \log \prod_{h=1}^{H} p(y_{nh,l}|z_{n,l} = k; a_{hk}, b_{hk})
\end{aligned} \tag{A4}$$

where the last equality follows from Eq. (A3). The expectation operation (denoted by $\mathbb{E}$) indicated in the first equality of Eq. (A4) is based on the joint posterior probability distribution on the latent state trajectory estimated using the last best estimate ($\widehat{\Theta}$) of the HMM. In the M-step, we determine the values for $\{\boldsymbol{\pi}_l\}_{l=1}^{L}$, $\boldsymbol{A}$ and $\boldsymbol{\phi}$ that maximize $Q(\Theta, \widehat{\Theta})$ in Eq. (A4), which we then use to update $\widehat{\Theta}$ (Sec A4). Additional constraints on this maximization problem are given by the equality constraints on $\boldsymbol{A}$ (Eq. (4) in main text) and $\boldsymbol{\pi}$ (Eq. (5) in main text), and the following inequality constraints: $A_{jk} \geq 0$, $\pi_k \geq 0$, $a_{hk}^2 + b_{hk}^2 - 2 > 0$ for all $j, k \in [1, K]$ and $h \in [1, H]$. The last set of inequality constraints on the beta pdf parameters leads to unimodal beta pdf's.

For an exponential family of observation distributions, the sequence of $\widehat{\Theta}$'s, resulting from maximizing $Q(\Theta, \widehat{\Theta})$ in each EM iteration, leads to a monotonic approach to a maxima of the likelihood function [3, 6–8]. Therefore, we use the estimated log-likelihood to track EM-algorithm convergence, i.e. we terminate the EM algorithm at an iteration number, t, when $p(\{\boldsymbol{Y}_1, \cdots, \boldsymbol{Y}_L\}|\widehat{\Theta}^{(t)}) - p(\{\boldsymbol{Y}_1, \cdots, \boldsymbol{Y}_L\}|\widehat{\Theta}^{(t-1)})) < 0.0001$. Since the EM algorithm is known to converge to local maxima [8], we repeat the EM algorithm with multiple initial guesses of Θ and choose the set of model parameters with the highest likelihood. Essential steps relevant to the M-step are presented in Sec A4.

Furthermore, using the ML beta-HMM we solve for the optimal state trajectory $\{z_{n,l}^{\star}\}_{n=1}^{N_l} = \arg\max_{\boldsymbol{Z}_l} p(\boldsymbol{Y}_l, \boldsymbol{Z}_l|\widehat{\Theta})$ using the *Viterbi* algorithm [2, Sec. 13.2]. This state trajectory represents the unsupervised segmentation of the data sequence, $\boldsymbol{Y}_l$. The output of the HMM fitted to the multiple observations from multiple sessions are the estimates of the ML parameter set, $\widehat{\Theta}$, and corresponding sequences of $\{\{\boldsymbol{\gamma}_{n,l}$, $\boldsymbol{\xi}_{n,l}$, $z_{n,l}^{\star}\}_{n=1}^{N_l}\}_{l=1}^{L}$. Since the assignment of state label is random, we re-number the states from 1 to K in the ascending order of the mean ($= a_{Hk}/(a_{Hk} + b_{Hk})$) of the beta distributions corresponding to the H-th

frequency band.

A4 M-step of EM

We calculate $\Theta^{(n)}$ by solving the constrained maximization of the $Q(\Theta, \widehat{\Theta})$ (Eq. (A4)) ,

$$\max_{\Theta} Q(\Theta, \widehat{\Theta}) \text{ such that,} \tag{A5}$$

Equality constraints:

$$\sum_{k=1}^{K} A_{jk} = 1 \; \forall\, k \in [1, K] \tag{A6}$$

$$\sum_{k=1}^{K} \pi_{k,l} = 1 \; \forall\, l \in [1, L], \tag{A7}$$

Inequality constraints:

$$-\pi_{k,l} \leq 0 \; \forall\, k \in [1, K],\; l \in [1, L] \tag{A8}$$

$$-A_{jk} \leq 0 \;;\; \forall\, j, k \in [1, K] \tag{A9}$$

$$-(a_{hk}^2 + b_{hk}^2 - 2) \leq 0 \, \forall\, h \in [1, H],\; k \in [1, K] \tag{A10}$$

Due to the independent additive contributions to the objective function due to each group of decision variables, $\pi_{k,l}$, $\boldsymbol{A}$ and $\{a_{hk}, b_{hk}\}$, we seek to increase $Q(\Theta, \widehat{\Theta})$ by maximizing these individual contributions independently. To implement this we solve the following optimization problems,

$$\widehat{\boldsymbol{\pi}}_l = \arg\max_{\boldsymbol{\pi}_l} \sum_{k=1}^{K} \gamma_{1,l}(k) \log \pi_{k,l} \;\text{, such that } \sum_{k=1}^{K} \pi_{k,l} = 1,\; \pi_{k,l} \geq 0 \tag{A11}$$

$$\begin{aligned} \{\widehat{A}_{jk}\}_{k=1}^{K} &= \arg\max_{\{A_{jk}\}_{k=1}^{K}} \sum_{l=1}^{L} \sum_{n=1}^{N_l} \sum_{j=1}^{K} \sum_{k=1}^{K} \xi_{n,l}(j,k) \log A_{jk} \\ &= \arg\max_{\{A_{jk}\}_{k=1}^{K}} \sum_{l=1}^{L} \sum_{n=1}^{N_l} \sum_{k=1}^{K} \xi_{n,l}(j,k) \log A_{jk} \\ &\text{such that } \sum_{k=1}^{K} A_{jk} = 1,\; A_{jk} \geq 0 \end{aligned} \tag{A12}$$

$$\begin{aligned} \{\widehat{a}_{hk}, \widehat{b}_{hk}\} &= \arg\max_{\{a_{hk}, b_{hk}\}} \sum_{l=1}^{L} \sum_{n=1}^{N_l} \sum_{k=1}^{K} \sum_{h=1}^{H} \gamma_{n,l}(k) \log Pr(y_{nh,l} | z_{n,l} = k; a_{hk}, b_{hk}) \\ &= \arg\max_{\{a_{hk}, b_{hk}\}} \sum_{l=1}^{L} \sum_{n=1}^{N_l} \gamma_{n,l}(k) \log Pr(y_{nh,l} | z_{n,l} = k; a_{hk}, b_{hk}) \;\;, \\ &\text{such that } a_{hk}^2 + b_{hk}^2 - 2 > 0 \end{aligned} \tag{A13}$$

Note that in Eqs. (A11), (A12) and (A13) we are solving for 1, K and KH independent optimization

problems. The solutions to Eq. (A11) and the (A12) have the following closed-form expressions,

$$\widehat{\pi}_{k,l} = \gamma_{1,l}(k)\,, \tag{A14}$$

$$\widehat{A}_{jk} = \frac{\sum_{l=1}^{L}\sum_{n=1}^{N_l}\xi_{n,l}(j,k)}{\sum_{k=1}^{K}\sum_{l=1}^{L}\sum_{n=1}^{N_l}\xi_{n,l}(j,k)} \tag{A15}$$

To solve the constrained optimization problem in Eq. (A13), we use the Lagrange multiplier method [9]. Alternatively, we can directly employ numerical tools for constrained optimization such as the `fmincon` function in Matlab.

A5 Algorithm to simulate a spectrogram generated by a known Markov process

In order to test the beta-HMM estimation framework, we simulated spectrograms generated by a pre-specified Markov sequence (see algorithm in Table A2). The inputs to the algorithm are a spectrogram estimated from one session of NHP experimental data (Sec 3.2.2), the number of states K to be simulated, the duration of the spectrogram to be simulated M, and the Markov process' parameters $\boldsymbol{\pi}$ and $\boldsymbol{A}$. The output of the algorithm is the Markov state path, $\boldsymbol{z} = \{z_m\}_{m=1}^{M}$, corresponding simulated spectrogram $\boldsymbol{S}^{sim}(\{\omega_i\}) = \{S_m^{sim}(\{\omega_i\})\}_{m=1}^{M}$ and scaled observation sequence $\boldsymbol{Y}$, and the beta distribution parameters associated with each state $\boldsymbol{\phi}$.

Table A2: Algorithm to simulate data from an NHP LFP session with an underlying Markovian latent state path for numerically testing the beta-HMM framework

**Input**	Spectrogram $\{S_n(\omega_i)\}_{n=1}^{N}$ (for every frequency $\omega_i \in \{\omega_r, 2\omega_r, \cdots, 50\}$) from one LFP recording session from NHP MJ of duration 20 min, user-specified number of states K and HMM parameters $\boldsymbol{\pi}$ and $\boldsymbol{A}$, and length of session to simulate, $M = 12000$.
1	Calculate $\{\bar{S}_n(\boldsymbol{\omega})\}_{n=1}^{N}$ (Eq. A1).
2	Identify K distinct clusters of spectra by executing a k-means clustering algorithm ([2]) on $\{\bar{S}_n(\boldsymbol{\omega})\}_{n=1}^{N}$. Label each cluster from 1 to K in ascending order of within-cluster mean power in the 35-50 Hz (h = 7) frequency band. Note: Each time-point n is now associated with a cluster label 1 to K.
3	Store the original time-point indices corresponding to each element in k-th cluster in an integer-valued set $\boldsymbol{n}_k$, where $k \in [1, K]$. Note: $\bigcup_{k=1}^{K} \boldsymbol{n}_k = [1, N]$ and $\bigcap_{k=1}^{K} \boldsymbol{n}_k = \varnothing$, where $\varnothing$ denotes the null set.
4	Simulate a state path by generating a Markov sequence $\{z_m\}_{m=1}^{M}$ using the set of parameters $\{\boldsymbol{\pi}, \boldsymbol{A}\}$.
5	Simulate an LFP spectrogram, $\{S_m^{sim}(\cdot)\}_{m=1}^{M}$, as follows: when $z_m = k$, uniformly sample index n_k from $\boldsymbol{n}_k$, and set $S_m^{sim}(\omega_i) = S_{n_k}(\omega_i)$ for every frequency $\omega_i \in \{\omega_r, 2\omega_r, \cdots, 50\}$. Also, set the frequency band-specific average of the simulated spectrum corresponding to $z_m = k$, $\bar{S}_m^{sim}(\boldsymbol{\omega}) = \bar{S}_{n_k}(\boldsymbol{\omega})$.
6	Calculate $\boldsymbol{Y} = \{\boldsymbol{y}_m\}_{m=1}^{M}$ using $\{\bar{S}_m^{sim}(\boldsymbol{\omega})\}_{m=1}^{M}$ (Eq. (1) in main text).
7	Determine cluster-specific and frequency-band specific maximum-likelihood estimate of the beta distribution parameters, $\{a_{hk}, b_{hk}\}$, for all $h = \{1, 2, ..., H\}$ and $k = \{1, 2, ..., K\}$ from $\boldsymbol{Y}$. Note: $\boldsymbol{\phi} = \{\{a_{hk}, b_{hk}\}_{h=1}^{H}\}_{k=1}^{K}$ estimated based on the simulated state path is considered as *ground truth* for the purpose of numerically testing the beta-HMM estimation framework.
**Output**	$\{S_m^{sim}(\omega_i)\}_{m=1}^{M}$ (for every frequency $\omega_i \in \{\omega_r, 2\omega_r, \cdots, 50\}$) and corresponding $\boldsymbol{Y}$, $\{z_m\}_{m=1}^{M}$, and $\boldsymbol{\phi}$.

A6 Tutorial on analyzing HMM state-specific spectra in terms of corresponding estimated beta pdf's

Here we present a tutorial-styled discussion on how to use HMM state-specific frequency band-specific beta pdf's (estimated using EM algorithm described in Sec A3) to interpret the corresponding HMM state-specific spectral profiles. To demonstrate the utility of the state-specific beta distributions, we use segmented data from the 2 HMM states, states 2 and 5, identified in the single session analysis of NHP MJ LFP data (Fig 3 in main text). To elucidate the correspondence between the spectral profile (Fig A1A, A1B, A1E, and A1F) and the respective empirical pdfs of the scaled powers (Fig A1C and A1G), we recall the intuition provided in Sec 3.1.2 with regard to the scaling equation (Eq.(1) in main text). Recall that the scaling factor for each frequency band is calculated from the quartiles of the spectrogram across the entire session. As a result, if the distribution of spectral power corresponding to a given state and frequency band has a similar variance to that of the entire session, its scaled representation will have high variance. This will correspond to a relatively flat beta distribution. In contrast, if the spectral power from a given state and frequency band tends to be greater than the median of the corresponding frequency band, the scaled observations will be skewed towards 1. Particularly, if the observations corresponding to a given HMM state are mostly between the third and fourth quartile, the pdf of the beta distribution for that state will have high probability mass between 0.75 and 1. Note that corresponding statements can be made for states with relatively lower spectral power in a given frequency band (i.e., the beta distributions would be skewed towards 0). We use this intuition to quantitatively characterize the spectral power in a given frequency band for states 2 and 5.

As seen in Fig A1A and A1B, there is prominent power in the 0-1 ($h = 1$), 1-4 ($h = 2$), 4-8 ($h = 3$), and 8-12 ($h = 4$) Hz bands for state 2. These power values are higher than the median value across the entire session, as indicated by high probability mass between 0.5 and 1 in Fig A1C. This right shift of the probability mass towards 1 in the scaled power pdf's is also represented by a high complementary cdf (ccdf – at a given sample point, ccdf equals 1 minus the value of the cdf at the same sample point) at 0.5 (Fig A1D) with $Pr(Y_1 > 0.5|Z = 2) = 1.00$, $Pr(Y_2 > 0.5|Z = 2) = 1.00$, $Pr(Y_3 > 0.5|Z = 2) = 1.00$ $Pr(Y_4 > 0.5|Z = 2) = 0.93$. In state 2, there is not much activity in the 12-25 Hz band (Fig A1B). This is similar to the activity across the majority of the session (Fig 3 in main text), and so the distribution of the scaled power has high variance (Fig A1C). In state 2, there is relatively less activity in the 25-35Hz, and 35-50 Hz bands (Fig A1B), and the power values are mostly lower than the median (as indicated by high probability mass below 0.5 in Fig A1C). However, note that there is high variance in the gamma bands in state 2, and so the distributions are relatively flat. The left shift of the probability mass of scaled power is also represented by the low ccdf values at 0.5 (Fig A1D) with $Pr(Y_6 > 0.5|Z = 2) = 0.37$, and $Pr(Y_7 > 0.5|Z = 2) = 0.24$.

Now using the intuitions built from our discussion of state 2, let us try to interpret the spectral representation of state 5. Compared to state 2, state 5 has lower activity in the 0-1 band (Fig A1E and A1F). However, compared to the pre-ketamine state (See state 1 in Fig 3 in main text), there is still significant activity in these bands. Thus, the scaled power in the 0-1 band for state 5 tends to be centered around the median power of the entire session (Fig A1G). This is reflected by a ccdf value near 0.5 ($Pr(Y_1 > 0.5|Z = 5) = 0.55$, Fig A1H). State 5 also tends to have activity in the 1-4, 4-8, and 8-12 Hz frequency bands, but it is more variable than in state 2. This results in right skewed, relatively flat scaled power distributions. In the gamma (25-35 and 35-50 Hz) bands there is prominent activity (Fig A1E and A1F), and the large probability mass near 1 in the scaled power pdf's (Fig A1G) indicates that these activities are higher than the respective

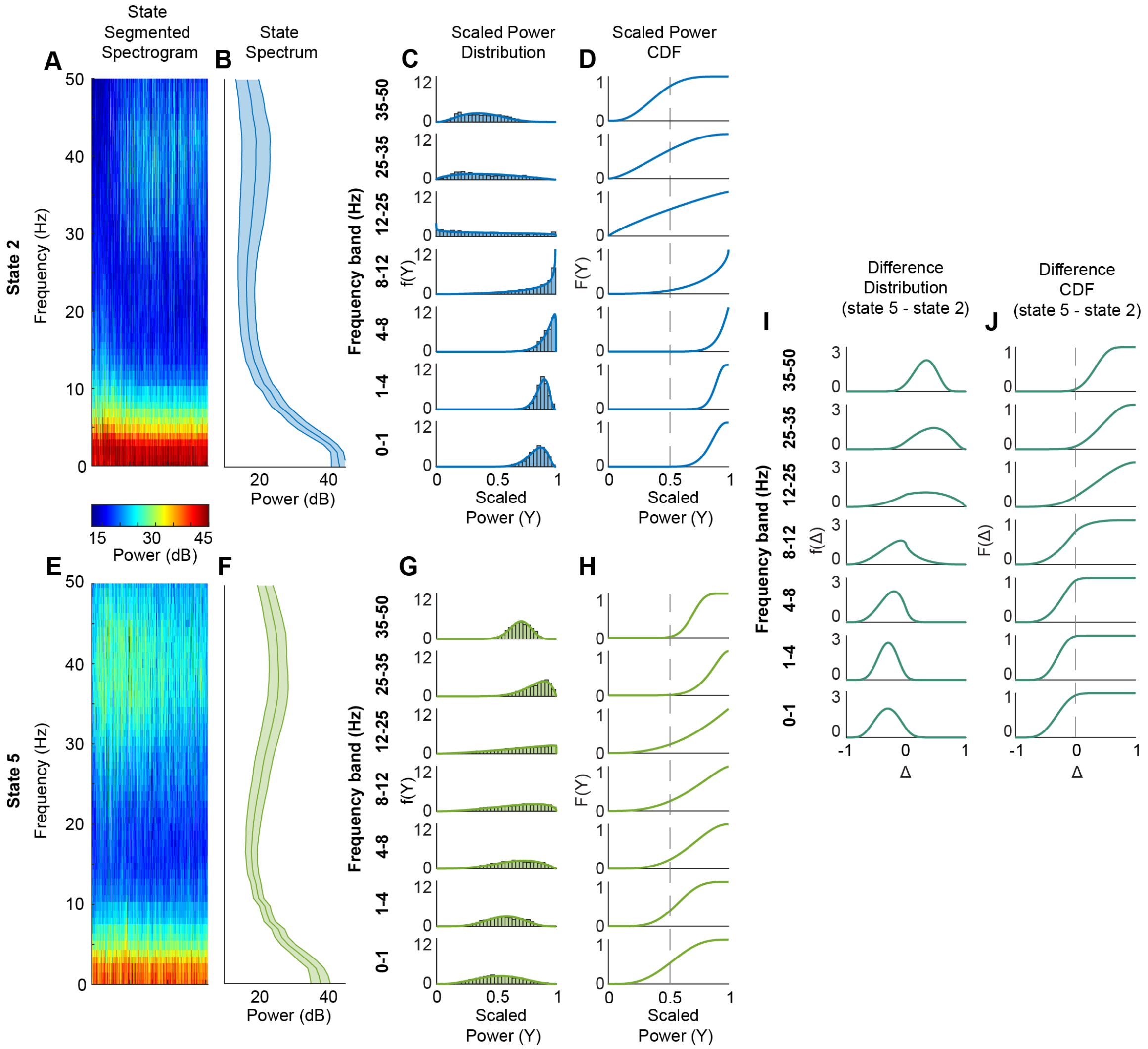

Figure A1: **Illustrative example of how to interpret the state-specific spectral profiles in terms of the state-specific frequency-band specific beta pdf's.** (A, E) Group of spectra from time-points identified as states 2 and 5 (by the Viterbi algorithm), respectively, of a 5 state beta-HMM fitted to a single session of NHP MJ data. (B,F) The average and standard deviation across all the spectral profiles for states 2 and 5, respectively. (C, G) Empirical pdfs of the scaled power in the 7 frequency bands, based on the data from the time-points identified as states 2 and 5 (by the Viterbi algorithm), respectively. The state-specific frequency-band specific beta pdfs of the maximum likelihood beta-HMM (estimated by the EM algorithm) is overlaid. (D, H) The cdf corresponding to the estimated beta pdfs plotted in C and G, respectively. (I) The frequency-band specific pdf of the difference random variable $\Delta = X_{h,5} - X_{h,2}$, where $X_{h,5} \sim Beta(a_{h,5}, b_{h,5})$ and $X_{5,h} \sim Beta(a_{h,2}, b_{h,2})$ based on the respective estimated pdfs, (J) The cdf corresponding to the frequency band-specific pdf's in (I).

median values. This right shift is clearly captured by the ccdf values at 0.5 – $Pr(Y_6 > 0.5|Z = 5) = 0.99$, and $Pr(Y_7 > 0.5|Z = 5) = 0.98$.

We can further use these beta pdfs to quantitatively compare the power values in a given frequency band between states 2 and 5. Using Eq. (6), we define a $\Delta_{h,5,2} = X_{h,5}–X_{h,2}$, such that $X_{h,5} \sim Beta(a_{h,5}, b_{h,5})$ and $X_{h,2} \sim Beta(a_{h,2}, b_{h,2})$. Figs A1I and A1J represent the pdf and cdf for the $\Delta_{h,5,2}$ where $h \in [1, 5]$. For the 0-1 ($h = 1$), 1-4 ($h = 2$), and 4-8 ($h = 3$) bands, most of the probability mass on $\Delta_{h,5,2}$ lies between -1 and 0, and the corresponding CDF values at 0 ($Pr(\Delta_{h,5,2} \leq 0)$) are 0.95, 0.99, and 0.94, respectively. This indicates that state 2 tends to have higher power in the low frequency bands compared to state 5. For the 8-12 ($h = 4$) and 12-25 ($h = 5$) Hz bands, the distributions are left and right skewed, respectively, but the probability mass on $\Delta_{h,5,2}$ is more evenly distributed around 0 ($Pr(\Delta_{4,5,2} \leq 0) = 0.74$, $Pr(\Delta_{h,5,2} \leq 0) = 0.23$). This indicates that these frequency bands are less likely to be significant discriminating factors between the two states. For the 25-35 ($h = 5$) and 35-50 ($h = 6$) bands, most of the probability mass of the respective Δ's occurs to the right of 0, which results in CDF values at 0 of 0.06 and 0.05, respectively. This indicates that the power in these two frequency bands is higher in state 5 compared to state 2. In summary, this analysis indicates that the activities in the 0-1, 1-4, 4-8, 25-35, and 35-50 Hz bands are likely to be significant discriminating factors between the two states. Thus, we can generate a quantitative description to support our qualitative assessment that state 2 has higher low-frequency (slow-theta) power, and state 5 has higher high-frequency (gamma) power.

References

1. Rosenthal JS. First Look At Rigorous Probability Theory, A. World Scientific Publishing Company; 2006.
2. Bishop CM. Pattern recognition and machine learning. springer; 2006.
3. Rabiner LR. A tutorial on hidden Markov models and selected applications in speech recognition. Proceedings of the IEEE. 1989;77(2):257–286. doi:10.1109/5.18626.
4. Shumway RH, Stoffer DS. Time series analysis and its applications: with R examples. Springer; 2017.
5. Dempster AP, Laird NM, Rubin DB. Maximum likelihood from incomplete data via the EM algorithm. Journal of the Royal Statistical Society: Series B (Methodological). 1977;39(1):1–22.
6. Baum LE, Petrie T, Soules G, Weiss N. A maximization technique occurring in the statistical analysis of probabilistic functions of Markov chains. The annals of mathematical statistics. 1970;41(1):164–171.
7. Liporace L. Maximum likelihood estimation for multivariate observations of Markov sources. IEEE Transactions on Information Theory. 1982;28(5):729–734.
8. Wu CFJ. On the Convergence Properties of the EM Algorithm. Ann Statist. 1983;11(1):95–103. doi:10.1214/aos/1176346060.
9. Bertsekas DP. Constrained optimization and Lagrange multiplier methods. Academic press; 2014.
