# Supplementary figures and images for: A hidden Markov model reliably characterizes ketamine-induced spectral dynamics in macaque local field potentials and human electroencephalograms
Source: PLoS Comput Biol. 2021 Aug 18;17(8):e1009280. doi: 10.1371/journal.pcbi.1009280 (PMC8405019; doi:10.1371/journal.pcbi.1009280)

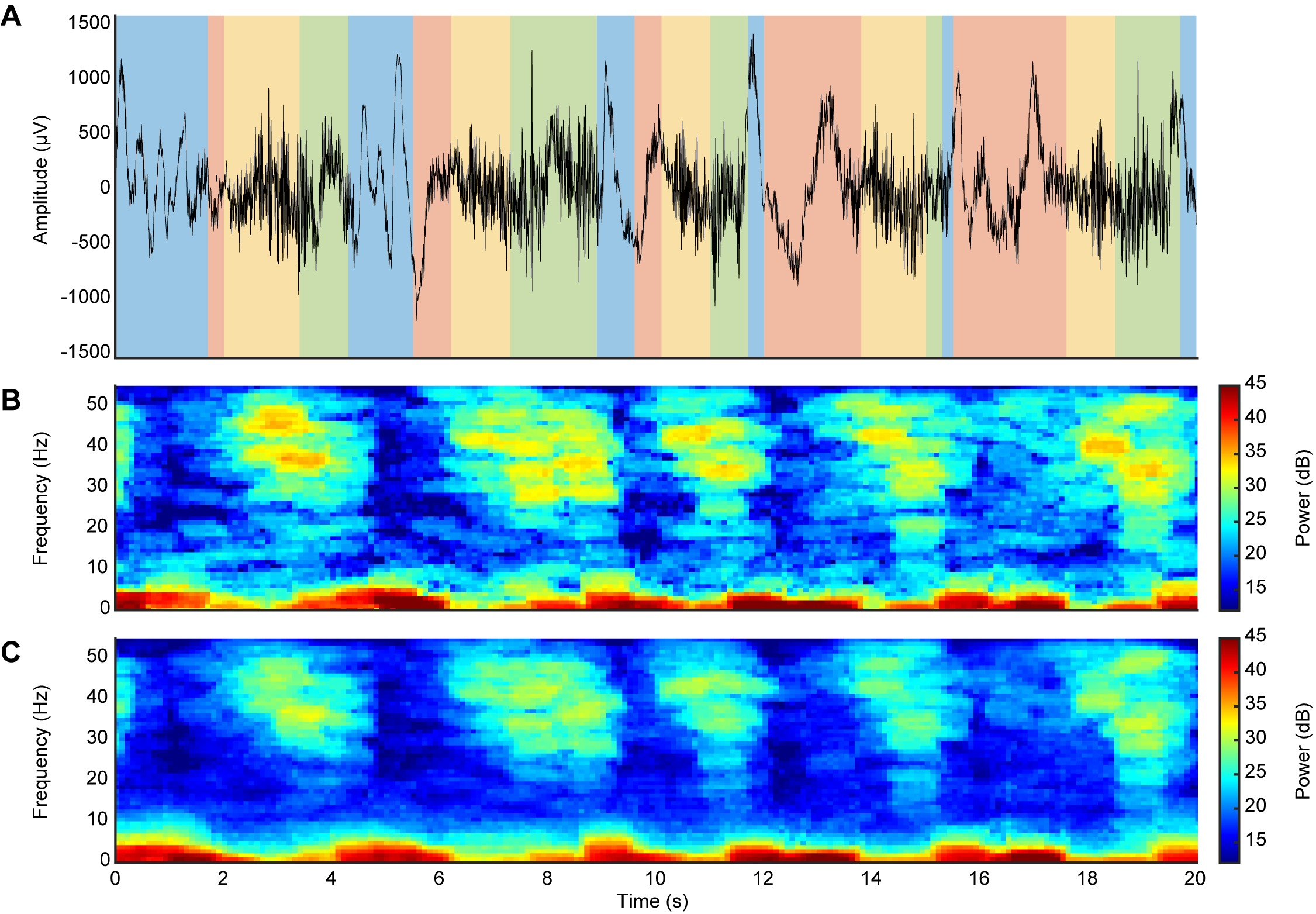

Supplement: S1 Fig — A typical 20 second epoch (following a high-dose ketamine bolus) of LFP from a single electrode of a multi-electrode array located in the frontal cortex (vlPFC) of NHP MJ is presented in panel A. Panel B shows the multitaper spectrogram for the same electrode, and panel C shows the average multitaper spectrogram for the vlPFC electrode array. Time 0 corresponds to 400 seconds after the ketamine bolus was administered. We fit our beta-HMM to the reduced-order representation of the corresponding spectrogram (derived using Eqs. (1) and (A1) in S1 Appendix), presented in panel C. The resulting optimal segmentation is represented by the colored vertical bars overlaid on the neural time-series in panel A. State 2 is shown in blue, state 3 in red, state 4 in yellow, and state 5 in green. (State 1, which corresponds to the time before the ketamine bolus, is not present in this epoch.). (TIF) [file pcbi.1009280.s002.tif]

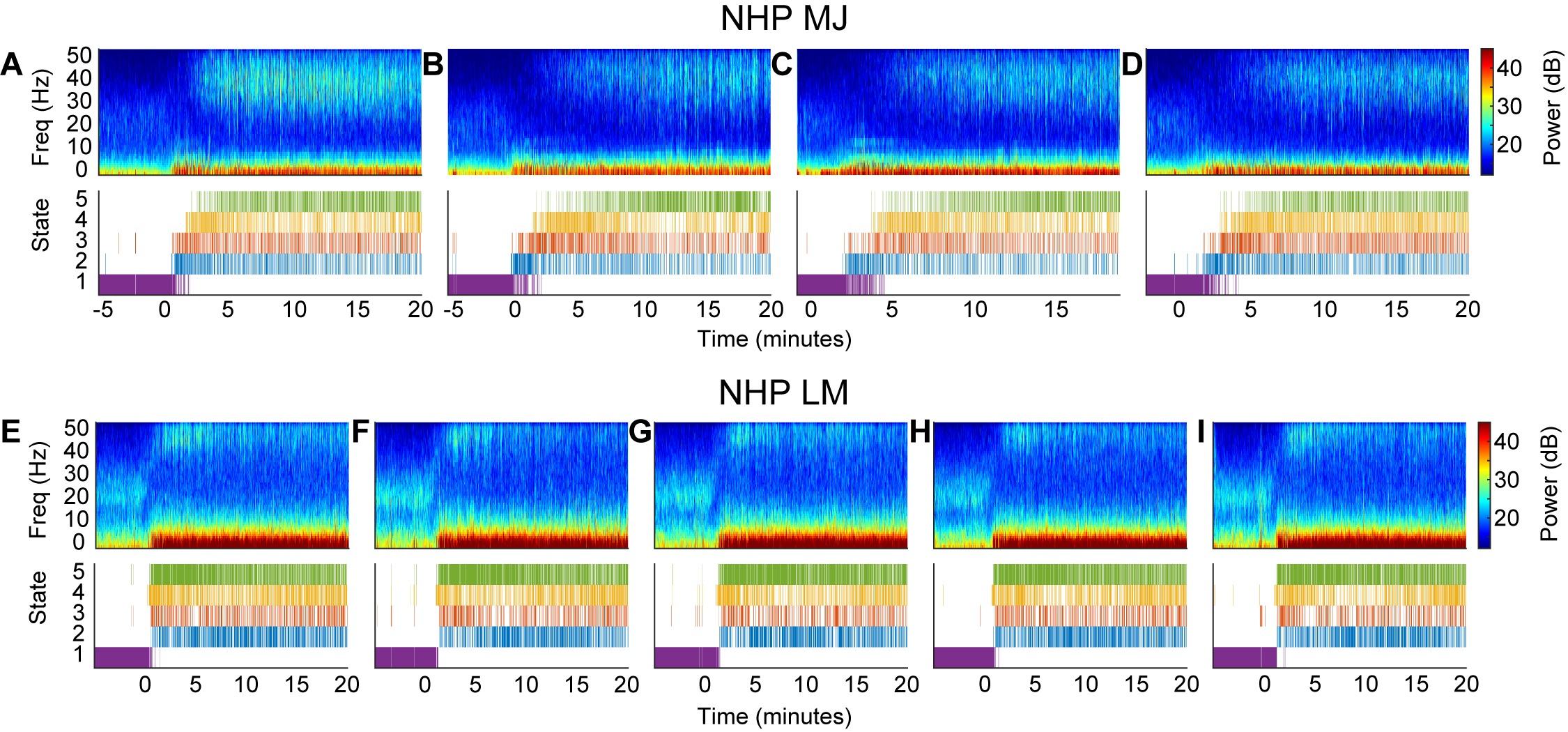

Supplement: S2 Fig — Multitaper spectrograms of LFP and corresponding estimated latent state trajectories from 4 sessions in NHP MJ (A-D) and 5 sessions in NHP LM (E-I). (TIF) [file pcbi.1009280.s003.tif]

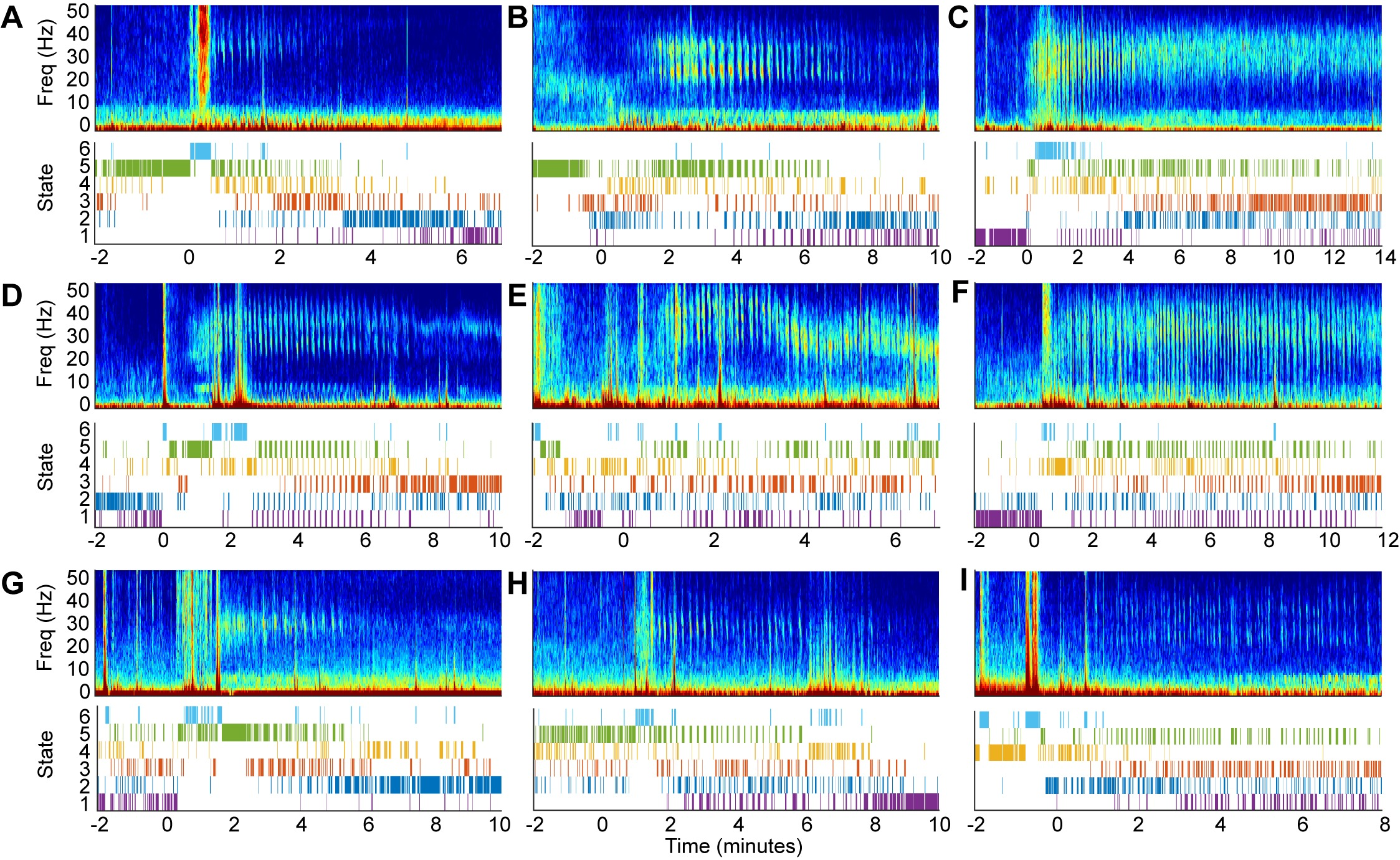

Supplement: S3 Fig — Multitaper spectrograms of EEG and corresponding estimated latent state trajectories from all 9 human subjects (A-I). Note that in many patients, the EEG activity before ketamine was administered is less reliably distinguished from the EEG activity after. However, in most patients (A, B, C, D, F, I) there is a clear change in the state trajectories after ketamine is administered. (TIF) [file pcbi.1009280.s004.tif]

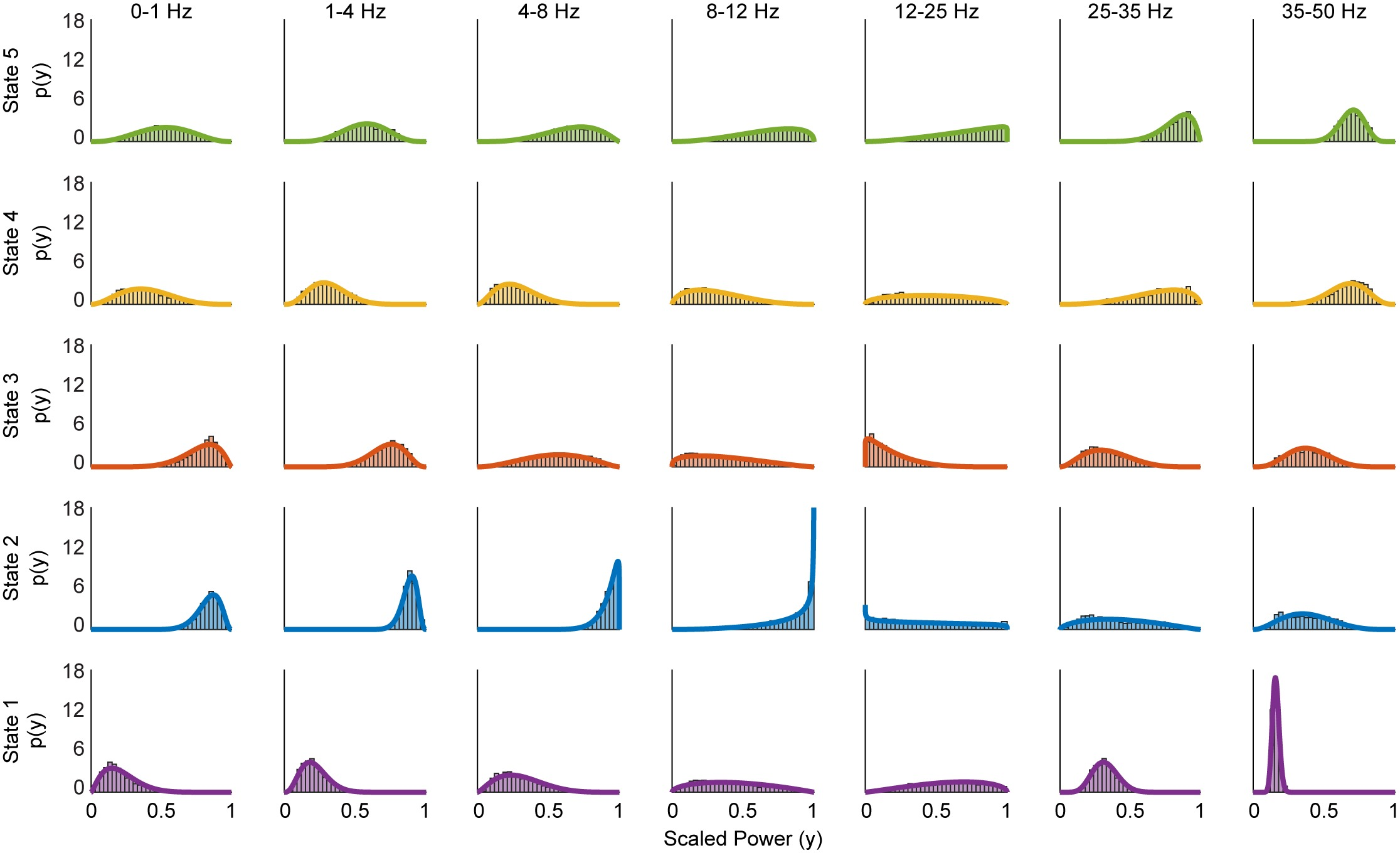

Supplement: S4 Fig — The state-specific and frequency-band specific beta pdfs corresponding to a 5-state beta-HMM were estimated from L = 1 session of LFP recording from NHP MJ. For each state (viewed row-wise) and a frequency band (viewed column-wise), the corresponding subplot presents (1) the empirical pdf plotted as a histogram based on the observations that correspond to the optimal segmentation of the data sequence, and (2) the continuous beta pdf with parameters estimated by the EM algorithm. Each color distinguishes a state as in Fig 3. (TIF) [file pcbi.1009280.s005.tif]

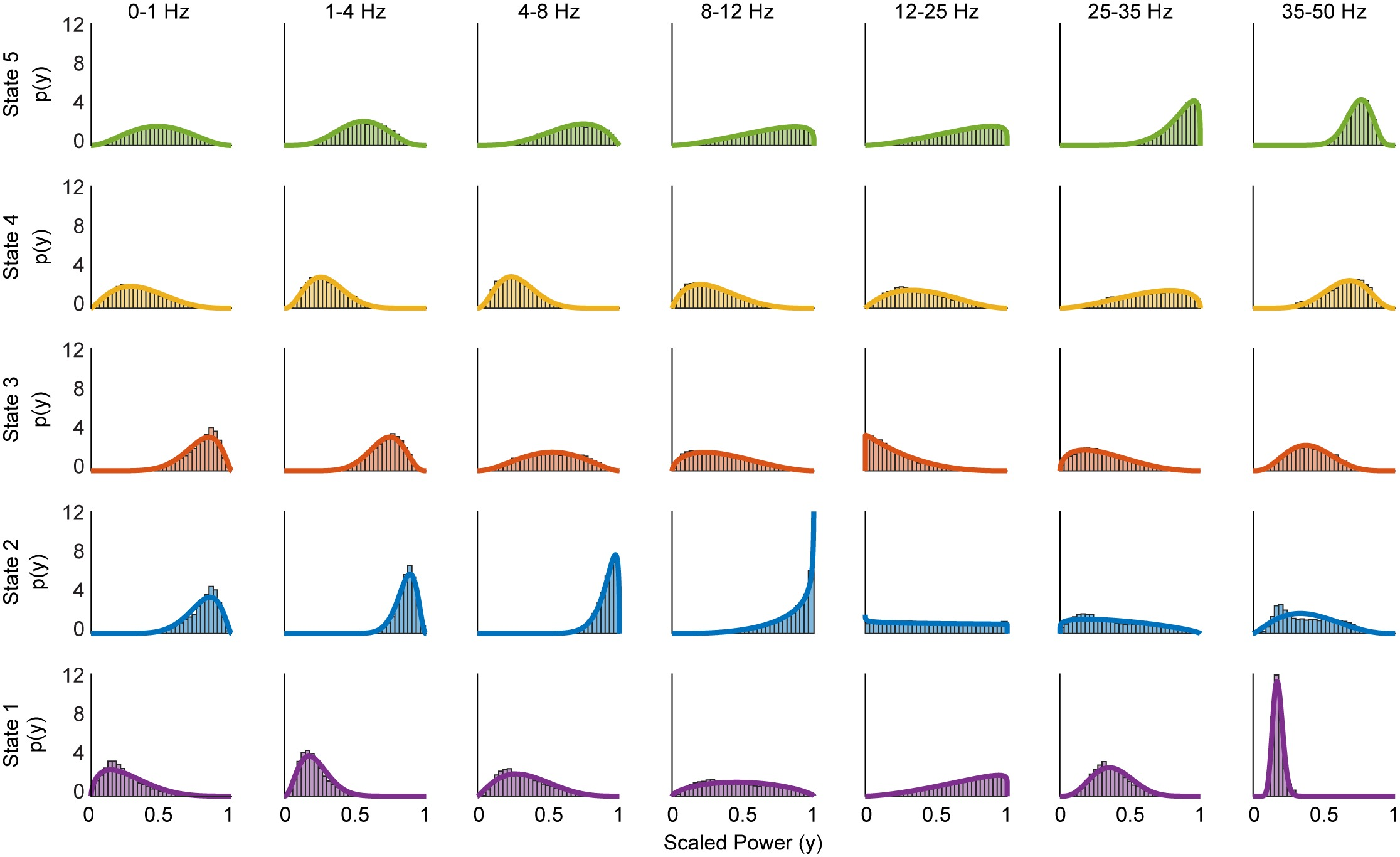

Supplement: S5 Fig — The state-specific and frequency-band specific beta pdfs corresponding to a 5-state beta-HMM were estimated from L = 4 sessions of LFP recording from NHP MJ. For each state (viewed row-wise) and a frequency band (viewed column-wise), the corresponding subplot presents (1) the empirical pdf plotted as a histogram based on the observations that correspond to the optimal segmentation (Viterbi algorithm) across the L = 4 sessions, and (2) the continuous beta pdf with parameters estimated by the EM algorithm. Each color distinguishes a state as in Fig 4. (TIF) [file pcbi.1009280.s006.tif]

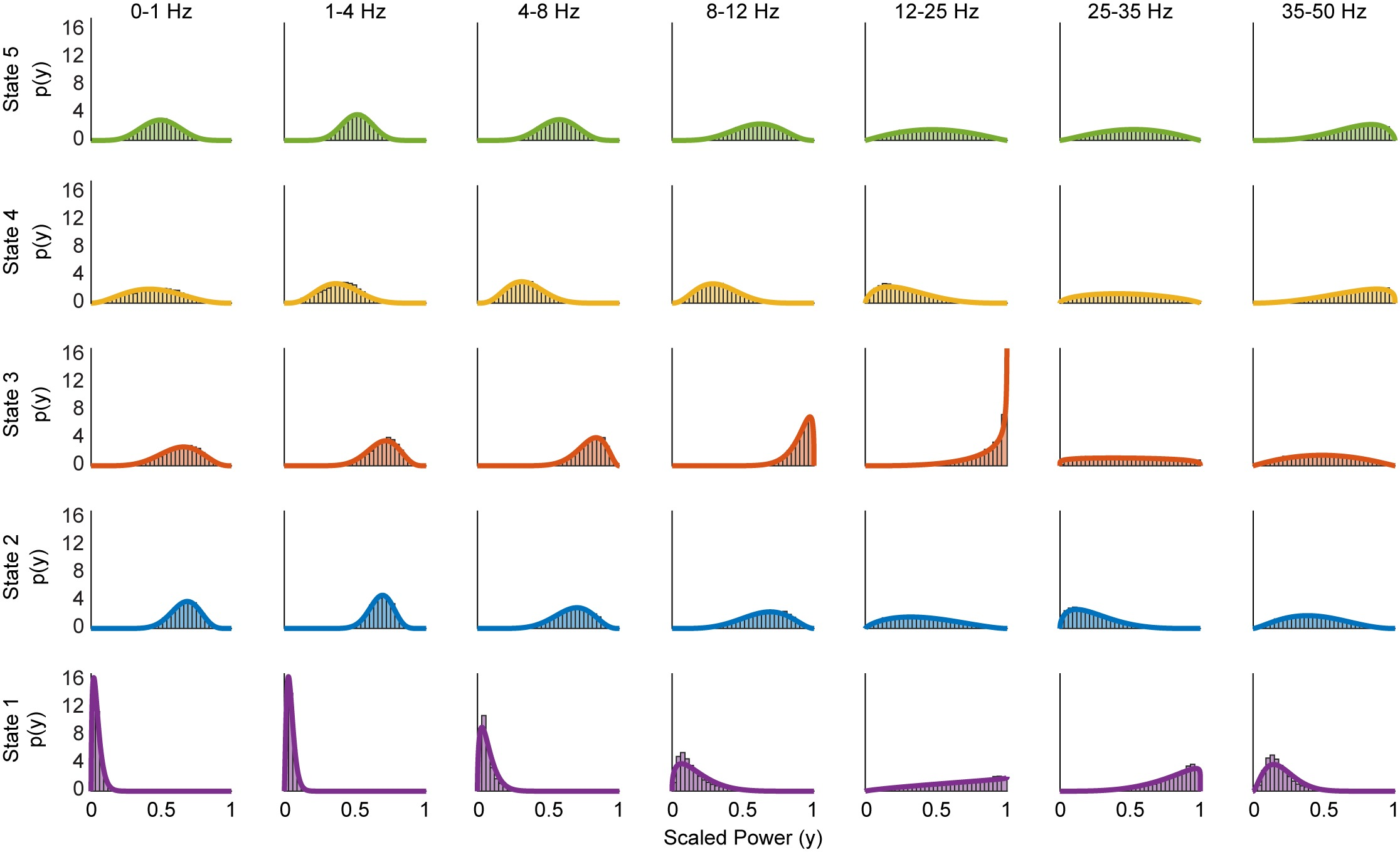

Supplement: S6 Fig — The state-specific and frequency-band specific beta pdfs corresponding to a 5-state beta-HMM were estimated from L = 5 sessions of LFP recording from NHP LM. For each state (viewed row-wise) and a frequency band (viewed column-wise), the corresponding subplot presents (1) the empirical pdf plotted as a histogram based on the observations that correspond to the optimal segmentation (Viterbi algorithm) across the L = 5 sessions, and (2) the continuous beta pdf with parameters estimated by the EM algorithm. Each color distinguishes a state as in Fig 4. (TIF) [file pcbi.1009280.s007.tif]

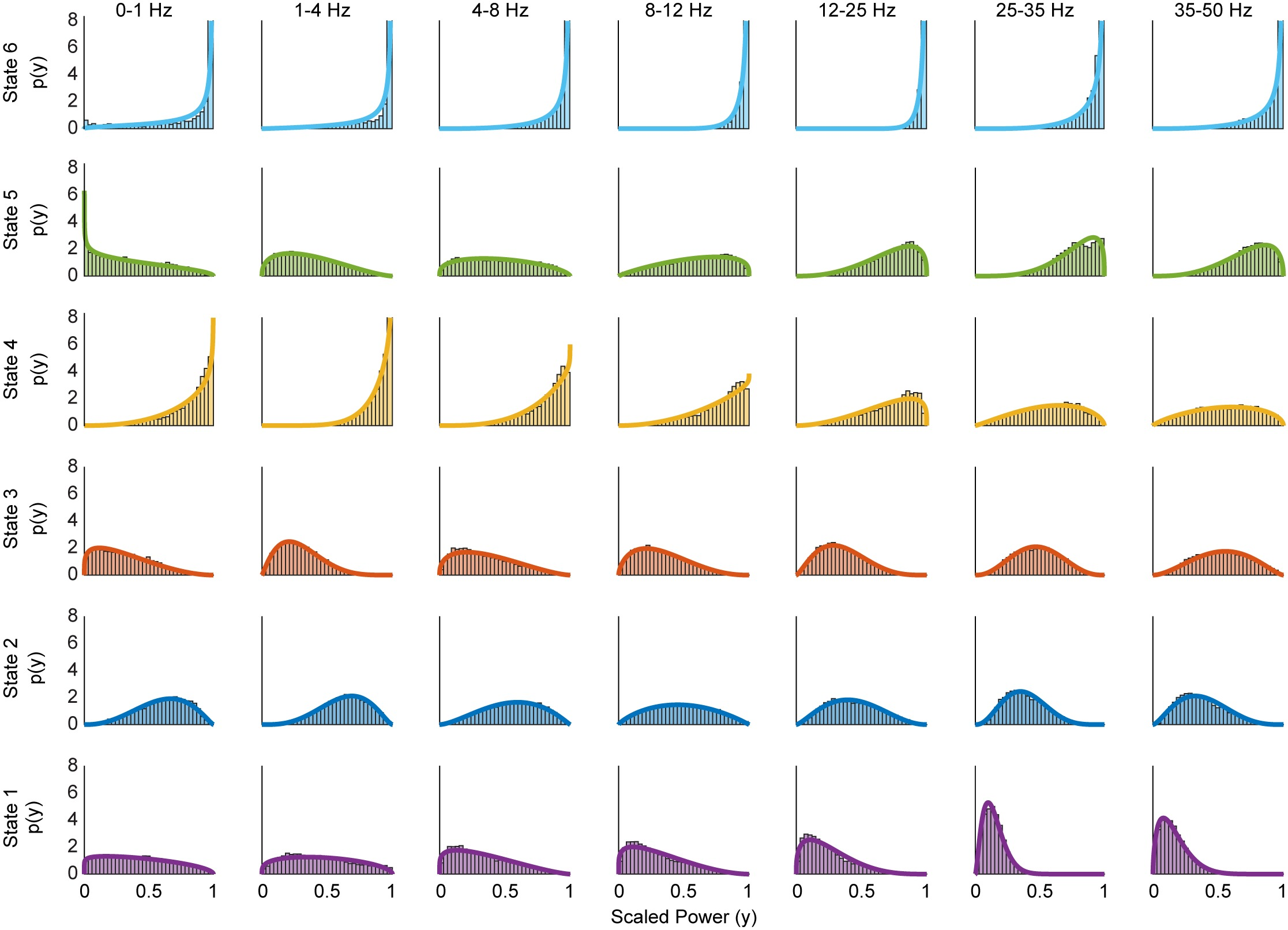

Supplement: S7 Fig — The state-specific and frequency-band specific beta pdfs corresponding to a 6-state beta-HMM were estimated from L = 9 sessions of EEG recording from 9 human OR patients. For each state (viewed row-wise) and a frequency band (viewed column-wise), the corresponding subplot presents (1) the empirical pdf plotted as a histogram based on the observations that correspond to the optimal segmentation (Viterbi algorithm) across the L = 9 sessions, and (2) the continuous beta pdf with parameters estimated by the EM algorithm. Each color distinguishes a state as in Fig 6. (TIF) [file pcbi.1009280.s008.tif]

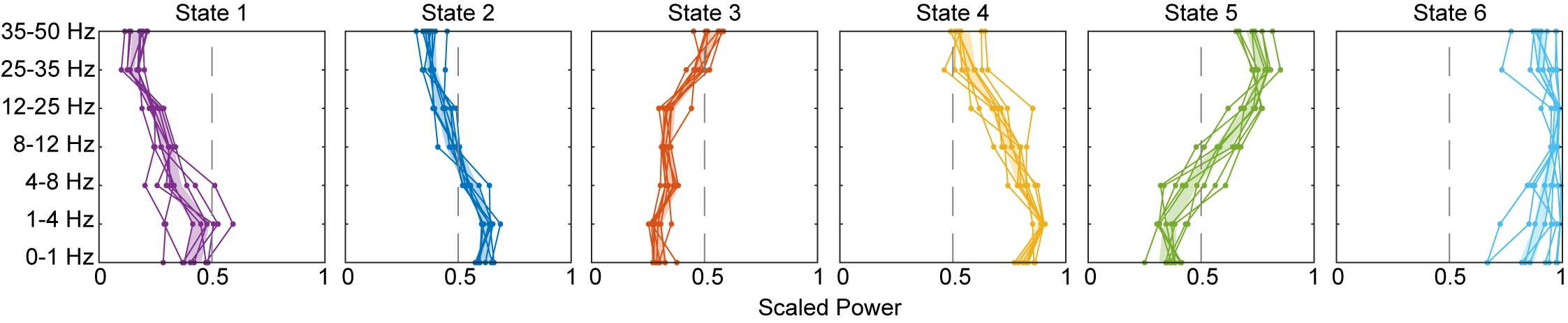

Supplement: S8 Fig — In each state plot, one dot represents the mean scaled power in a specific frequency band for one patient, where the mean is calculated from the observations assigned to that state via the Viterbi algorithm. The mean scaled power across frequencies for each patient are connected with a line. The shaded region indicates the 95% confidence interval for the mean scaled power across 10000 samples of the mean of the corresponding beta distribution, where the mean is calculated from 200 independent samples of the beta distribution. Note that while some observed means exceed the 95% confidence interval of the expected mean of the corresponding estimated beta distribution, the overall trends are consistent across patients. (TIF) [file pcbi.1009280.s009.tif]

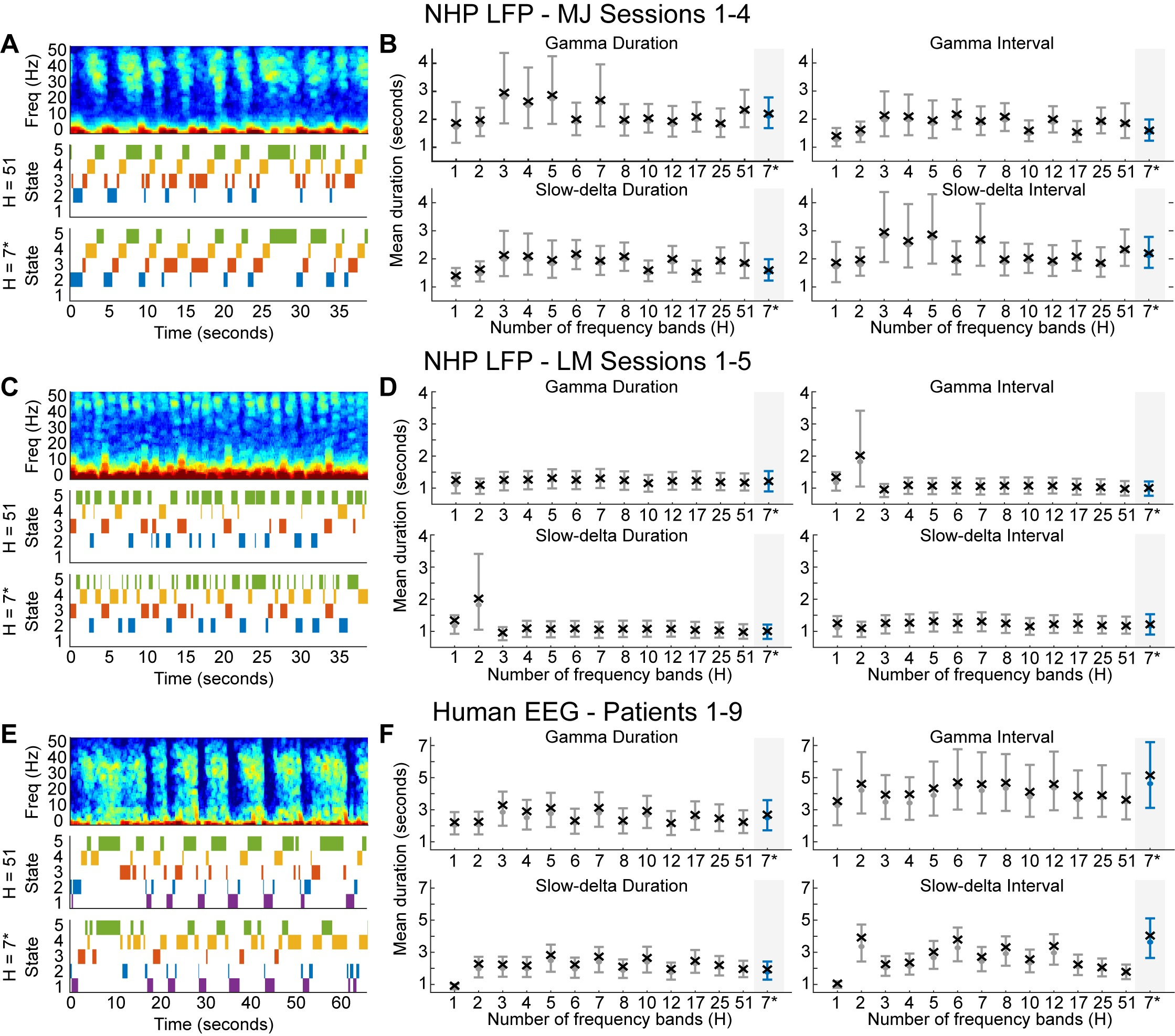

Supplement: S9 Fig — Panels A and B present analysis from NHP MJ sessions 1–4, panels C and D present analysis from NHP LM sessions 1–5, and panels E and F present analysis from the 9 human EEG sessions. In panels A, C, and E, a portion of the spectrogram is displayed on the top, and two state trajectories are displayed below, estimated from models with H = 51 frequency bands (i.e. the highest possible frequency resolution equal to that of the estimated spectrogram) and H = 7* frequency bands (where 7* frequency bands indicate the canonical frequency bands described in Sec 3.2.2). Panels B, D, and F present the key duration statistics presented in Sec 4.3 and 4.4 across varying frequency-band resolution. With the exception of the models corresponding to 7*, the canonical frequency bands, all other models utilized evenly spaced frequency bands. For each model, 4000 Markov sequences of length N = 2000 were simulated using the estimated transition matrix. The mean duration and interval corresponding to the gamma and slow-delta activities were calculated for each realization of these sequences. Median and 95% confidence bounds across simulated sequences for models with equally spaced frequency bands are indicated in grey and, for the model utilizing canonical frequency bands, in blue. The mean duration and interval calculated from the estimated state trajectory (output of the Viterbi algorithm which uses the maximum likelihood beta-HMM parameters and the observations as input) are indicated by a cross (×) symbol. Note that the 95% confidence intervals of the durations across the varying frequency resolutions almost always overlap. This indicates that the specification of the frequency bands does not have a critical effect on the inferences related to the model dynamics. Thus, in the main manuscript, we chose to present the model estimated from canonical frequency bands that are commonly used to describe neural oscillations. (TIF) [file pcbi.1009280.s010.tif]
